# A Hippocampus-Accumbens Tripartite Neuronal Motif Guides Appetitive Memory in Space

**DOI:** 10.1016/j.cell.2018.12.037

**Published:** 2019-03-07

**Authors:** Stéphanie Trouche, Vadim Koren, Natalie M. Doig, Tommas J. Ellender, Mohamady El-Gaby, Vítor Lopes-dos-Santos, Hayley M. Reeve, Pavel V. Perestenko, Farid N. Garas, Peter J. Magill, Andrew Sharott, David Dupret

**Affiliations:** 1 Medical Research Council Brain Network Dynamics Unit, Department of Pharmacology, University of Oxford, OX1 3TH Oxford, UK

**Keywords:** hippocampus, nucleus accumbens, memory, cell assembly, spatial representation, reward

## Abstract

Retrieving and acting on memories of food-predicting environments are fundamental processes for animal survival. Hippocampal pyramidal cells (PYRs) of the mammalian brain provide mnemonic representations of space. Yet the substrates by which these hippocampal representations support memory-guided behavior remain unknown. Here, we uncover a direct connection from dorsal CA1 (dCA1) hippocampus to nucleus accumbens (NAc) that enables the behavioral manifestation of place-reward memories. By monitoring neuronal ensembles in mouse dCA1→NAc pathway, combined with cell-type selective optogenetic manipulations of input-defined postsynaptic neurons, we show that dCA1 PYRs drive NAc medium spiny neurons and orchestrate their spiking activity using feedforward inhibition mediated by dCA1-connected parvalbumin-expressing fast-spiking interneurons. This tripartite cross-circuit motif supports spatial appetitive memory and associated NAc assemblies, being independent of dorsal subiculum and dispensable for both spatial novelty detection and reward seeking. Our findings demonstrate that the dCA1→NAc pathway instantiates a limbic-motor interface for neuronal representations of space to promote effective appetitive behavior.

## Highlights

• Neuronal and behavioral reinstatement of a memory trace are dissociable processes
• Spatial appetitive memory requires direct dCA1 inputs to two types of NAc neurons
• dCA1 exerts feedforward inhibition onto its postsynaptic NAc medium spiny neurons
• dCA1 organizes assemblies of co-active medium spiny neurons during memory retrieval

## Graphical Abstract

**Figure:**
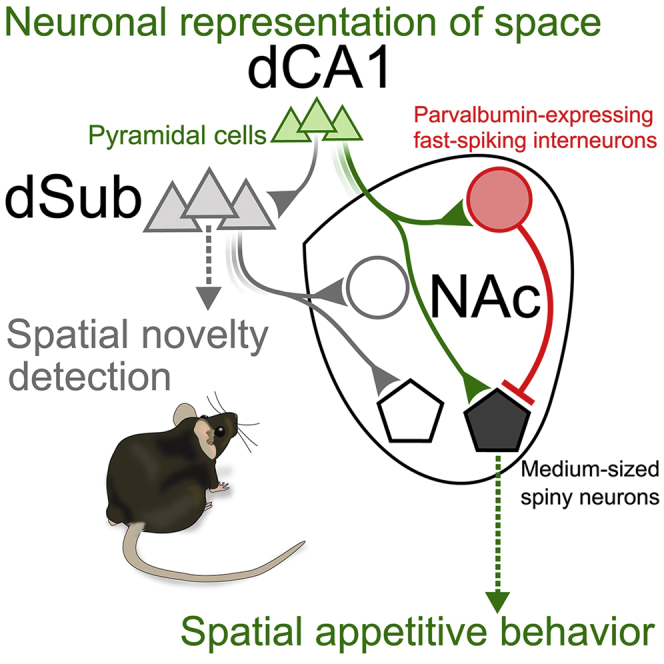

## Teaser

Retrieving and acting on memories of food-predicting environments are fundamental processes for animal survival. Trouche et al. show in mice that the behavioral manifestation of spatial appetitive memory requires a direct neural pathway from dorsal hippocampus to nucleus accumbens. This pathway engages dCA1 with a select population of accumbens parvalbumin-expressing fast-spiking interneurons, thereby sculpting the assembly activity of its postsynaptic medium-sized spiny neurons. An indirect pathway through dorsal subiculum is dispensable for appetitive memory but contributes to spatial novelty detection.

## Introduction

Directing spatial behavior toward environments predicting appetitive outcomes is fundamental for successful foraging and animal survival. In mammals, the expression of such adaptive behavior relies on the ability of the brain to make use of internal representations of space held in memory by pyramidal cells (PYRs) of the dorsal hippocampus (Moser et al., 2017, O’Keefe and Dostrovsky, 1971). Importantly, dorsal hippocampus PYRs do not have direct access to spinal and brainstem motor centers, and a comprehensive understanding of the substrates by which neuronal representations of space influence the behavioral expression of spatial appetitive memory is lacking.

The nucleus accumbens (NAc) of the ventral striatum has long been considered as an interface between the limbic and the motor systems and is important for processing cue-predicted reward outcomes and reward-motivated behavior (Carelli, 2002, Groenewegen et al., 1996, Mogenson et al., 1980, Pennartz et al., 1994). The NAc thus seems well placed to integrate dorsal hippocampal information about reward-predicting environments that can be used to guide appetitive behavior. Consistent with this idea, lesions and pharmacological manipulations altering NAc activity impair behavioral performance in spatial memory tasks that require the dorsal hippocampus (Annett et al., 1989, Ferbinteanu and McDonald, 2001, Ito et al., 2008, Schacter et al., 1989), suggesting that these two circuits are functionally connected. However, the nature and necessity of any link between dorsal hippocampus PYRs and NAc neurons remains unclear.

Using *in vivo* dual-site electrophysiological recordings, *ex vivo* slice physiology, viral vector-mediated tract tracing and ultra-structural anatomy, together with an intersectional *trans*-synaptic optogenetic strategy, this study unveils a tripartite neuronal motif linking the dorsal hippocampus to the NAc for guiding appetitive memory in space. In this motif, the direct recruitment of a subset of NAc parvalbumin-expressing fast-spiking interneurons (PV^+^FSIs) by PYRs from the CA1 region of the dorsal hippocampus (dCA1) enables the behavioral expression of neuronal representations of space and associated NAc assemblies of co-active medium-sized spiny neurons (MSNs). We show that dCA1 PYRs form functional synaptic contacts with both NAc MSNs and PV^+^FSIs and that this monosynaptic pathway is required for sucrose-conditioned place preference (CPP) memory. Both dorsal subiculum (dSub) inputs to NAc and those from dCA1 to dSub are dispensable for CPP memory retrieval. We establish that dCA1 PYRs support CPP memory by orchestrating the coincidental firing of postsynaptic MSN targets using a feedforward inhibition mechanism instantiated by a subset of PV^+^FSIs. Selectively silencing dCA1-connected PV^+^FSIs impairs the integration of dCA1 information by assemblies of co-active MSNs and disrupts CPP. Our findings identify a dCA1→NAc tripartite circuit motif engaging PYRs with both MSN and PV^+^FSI partners to behaviorally translate the neuronal reinstatement of a spatial appetitive memory.

## Results

### Two NAc Cell Types Receive dCA1 Monosynaptic Inputs

**Figure 1:**
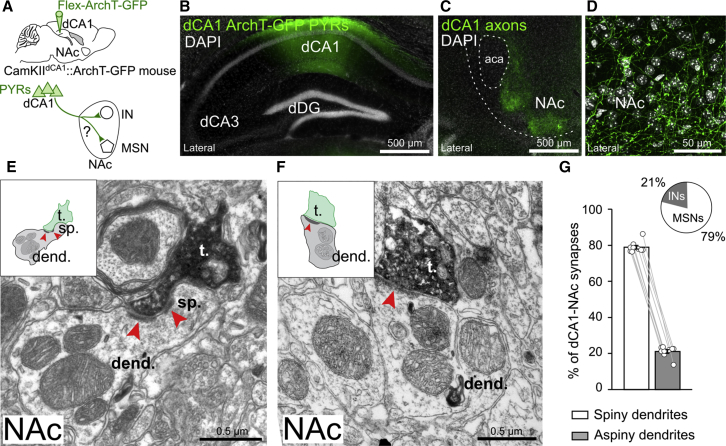
dCA1 Pyramidal Cells Form Monosynaptic Contacts with NAc Neurons (A) dCA1 PYRs of CamKII-Cre mice were transduced with ArchT-GFP, and their ArchT-GFP-expressing axons were examined in NAc. (B) Confocal image showing dCA1 ArchT-GFP expression in a CamKII^dCA1^::ArchT-GFP mouse. Cell nuclei stained with DAPI (gray). (C and D) dCA1 ArchT-GFP axons in NAc at a low (C) and a high (D) magnification. (E and F) Electron micrographs showing dCA1 ArchT-GFP axon terminals (labeled with DAB) making synaptic contacts (red arrowheads) with (E) the dendritic spine of an MSN and (F) a dendrite lacking spines in NAc. Schematic of synapses shown as insets. See also Figure S1. (G) Percentage of asymmetric synapses (mean ± SEM) formed between dCA1 terminals and either NAc spiny dendrites (of MSNs; 78.93% ± 0.99%) or aspiny dendrites (of putative interneurons; 21.07% ± 0.98%). Each data point represents a tissue block of NAc sections (n = 3 mice; 3 blocks per mouse; 353 ArchT-GFP dCA1-NAc synapses). dCA1, dorsal hippocampal CA1; dCA3, CA3; dDG, dentate gyrus; NAc, nucleus accumbens; PYRs, pyramidal cells; MSN, medium spiny neuron; IN, interneuron; aca, anterior commissure; t., dCA1 axon terminal; dend., dendrite; spine sp.; DAB, 3,3′-diaminobenzidine; ArchT, Archaerhodopsin-T.

**Figure S1:**
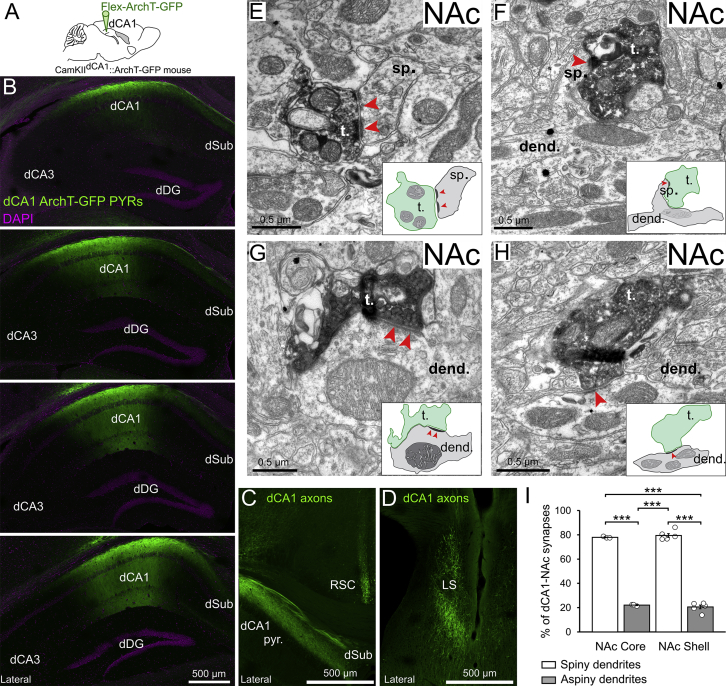
dCA1 Pyramidal Cells Innervate Both NAc MSNs and Putative Interneurons, Related to Figure 1 (A) dCA1 PYRs of CamKII-Cre mice were transduced with ArchT-GFP. (B) Serial confocal images from a CamKII^dCA1^::ArchT-GFP mouse. Note that the viral transduction of dCA1 PYRs did not spread to dSub neurons. (C and D) dCA1 ArchT-GFP axons observed in (C) dorsal subiculum and retrosplenial cortex, as well as (D) lateral septum. (E–H) Electron micrographs showing dCA1 ArchT-GFP axon terminals (labeled with DAB) making synaptic contacts (red arrowheads) with spiny dendrites of MSNs (E and F) and dendrites lacking spines of putative interneurons (G and H) in the NAc. Schematic of synapses shown as insets. See also Figures 1E and 1F. (I) The proportion of synapses formed by dCA1 terminals and either spiny or aspiny dendrites in NAc core and shell was not different (mean ± SEM). Each data point represents a tissue block of NAc sections. Both the proportion of dCA1 synapses formed with spiny dendrites and the proportion of dCA1 synapses formed with aspiny dendrites was similar in the shell and the core. See also Figure 1G. Abbreviations: dorsal hippocampal CA1 (dCA1), dorsal dentate gyrus (dDG), 3,3′-diaminobenzidine (DAB), nucleus accumbens (NAc), dorsal subiculum (dSub), retrosplenial cortex (RSC), lateral septum (LS), dCA1 axon terminal (t.), dendrite (dend.), spine (sp.), Green Fluorescent Protein (GFP).

To elucidate the neural substrates for a functional coupling between the dorsal hippocampus and the NAc, we first investigated whether dCA1 PYRs project to, and form synapses with, NAc neurons. We transduced dCA1 PYRs in CamKII-Cre mice with a Cre-inducible adeno-associated virus (AAV) vector carrying the yellow light (561 nm)-driven neural silencer Archaerhodopsin-T fused with the GFP reporter (ArchT-GFP) (Figures 1A, 1B, S1A, and S1B). In these CamKII^dCA1^::ArchT-GFP mice, we observed prominent expression of ArchT-GFP in axons located in the NAc (Figures 1C and 1D), as well as in other brain regions known to receive direct inputs from dCA1 (Figures S1C and S1D) (van Strien et al., 2009). Using electron microscopy, we found that dCA1 PYR axon terminals establish numerous asymmetric synapses (i.e., Gray’s type 1; typical of those formed by glutamatergic axons) with spiny dendrites of MSNs (Figures 1E, 1G, S1E, S1F, and S1I). The remaining dCA1 PYR axon terminals formed asymmetric synapses with dendrites that did not bear spines (Figures 1F, 1G, and S1G–S1I), which likely correspond to aspiny dendrites of NAc interneurons (Doig et al., 2010). The proportion of synapses formed with putative interneurons (∼20%; Figures 1G and S1I) was considerably larger than the proportion of NAc interneurons (< 5%) (Tepper et al., 2010), indicating an enriched innervation of this cell type. These data reveal the existence of a direct pathway from dCA1 PYRs to both NAc MSNs and interneurons.

### Spatial Appetitive Memory Requires dCA1 Assemblies

**Figure 2:**
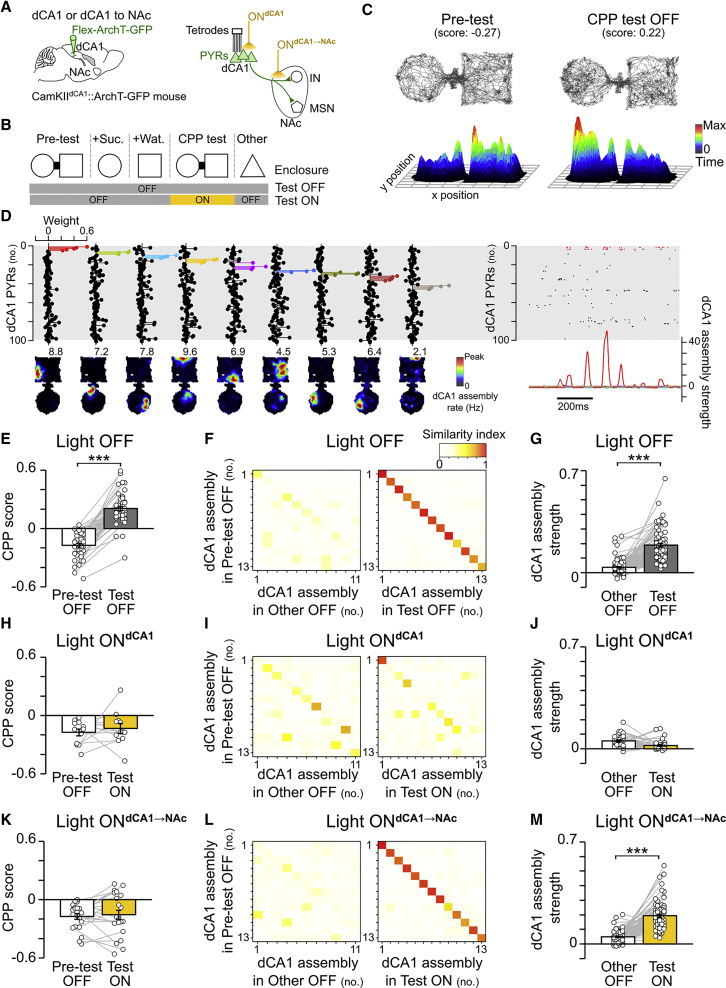
CPP Memory Requires Both dCA1 Representations and a Functional dCA1→NAc Pathway (A) dCA1 PYRs of CamKII-Cre mice were transduced with ArchT-GFP, and their firing activity was monitored with tetrodes. Delivery of yellow light to dCA1 somata (ON^dCA1^) or their axons in NAc (ON^dCA1→NAc^) was performed using optic fibers inserted above dCA1 or NAc, respectively. (B) Each day, mice performed a 1-day CPP task and explored an additional enclosure (other). In light ON days, yellow light was delivered during CPP test. (C) Example paths (top) and 3D occupancy maps (bottom) of a mouse performing the CPP task. CPP score equals time in sucrose-paired enclosure (+Suc.), minus that in water-paired enclosure (+Wat.), divided by the sum. A negative score indicates that the mouse spent less time in the enclosure paired with sucrose during conditioning. Note that during test, the mouse successfully changed its preference for the enclosure that was paired with sucrose, as indicated by the positive score. (D) Examples of dCA1 assembly representations of the CPP apparatus. For clarity, each assembly is depicted in the form of a weight vector where PYRs are ordered and color-coded in the lollipop plots to highlight those neurons with high coincidental spiking. Shown are the weight vectors (top-left) of the detected assembly patterns, the corresponding assembly maps (bottom-left; numbers indicate assembly activation rate), a sample raster plot of the spike trains (one PYR per row; top-right) and the corresponding activation strength time course of the detected assembly patterns (bottom-right; highlighting activation peaks of the red assembly). See also Figure S2. (E, H, and K) CPP score (mean ± SEM; n = 81 days from 21 mice) during pre-test and test (E: n = 48 light OFF; H: 12 ON^dCA1^; K: 21 ON^dCA1→NAc^). Light was delivered during CPP test (H: 20 × 30-s pulses over 15 min, K: one 10-min pulse). (F, I, and L) Example dCA1 assembly similarity matrices from 3 recording days. For clarity, the same number of assemblies is represented across those matrices. Note that in both light OFF (F) and ON^dCA1→NAc^ (L) conditions, the CPP assemblies were reinstated during test, but not during the exposure to the other (CPP-unrelated) enclosure, thus showing the context-dependent reinstatement of dCA1 representations. Reinstatement of dCA1 CPP representations was suppressed during ON^dCA1^ test (I; see also Figure S3). (G, J, and M) Activation strength of dCA1 CPP assemblies (mean ± SEM; n = 209 total detected assembly patterns; 11.61 ± 1.36 patterns per day; 1,176 PYRs total recorded from eight mice; 65.33 ± 7.91 PYRs per mouse recording day). Each pair of connected data points shows the average strength of a CPP assembly pattern tracked during other and test (G: n = 95 light OFF assemblies from 493 PYRs; J: 37 ON^dCA1^ assemblies from 227 PYRs; M: 77 ON^dCA1→NAc^ assemblies from 456 PYRs). ^∗∗∗^p < 0.001, paired t tests. See also Figure S4.

**Figure S2:**
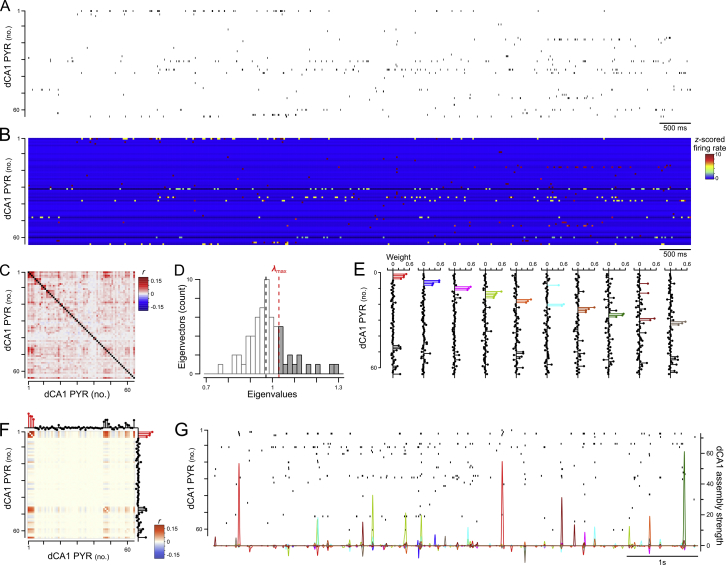
Detecting dCA1 Pyramidal Cell Assemblies and Tracking Their Activation Strength, Related to Figure 2 (A–E) Identification of dCA1 PYR assemblies. The different steps for the identification of assembly patterns formed by neuronal co-activation are illustrated for 63 simultaneously recorded pyramidal cells. The spike train of each recorded PYR (A) was binned (25ms time bins) and normalized (z-score). Principal component analysis (PCA) was next applied to the resulting correlation matrix (C) of that binned and *z*-scored spike-count matrix to find the number of patterns describing statistically significant co-activations between neurons, as estimated by the number of eigenvalues exceeding the Marcenko-Pastur threshold $\lambda_{\text{max}}$(D). Independent component analysis (ICA) was then used to identify the assembly patterns. The assembly patterns are represented in the form of lollipop weight vectors to indicate the contribution of each neuron to that pattern (E). For each pattern, neurons with a high contribution (i.e., weight exceeding two standard deviations above the mean) are colored for display purposes. For clarity, only a sample of few seconds of the spike trains raster plot (A) and the z-scored spike-count matrix (B) are shown. (F and G) Tracking of assembly pattern expression strength. For each assembly pattern, a projector matrix was constructed by taking the outer product matrix of its weight vector and setting the diagonal to zero (to ensure only co-activations of neurons can contribute to the expression of an assembly-pattern); (F) The spike trains from the recording sessions where the assembly patterns were tracked (i.e., CPP Test or Other; see Figures 2B, 2G, 2J, and 2M) and convolved with a Gaussian kernel. We estimated the assembly strength at each time point as the square of the projection of the z-scored (and convolved) population activity at that point onto a given independent component when removing the individual contribution of the neurons; (G) Assembly-pattern activations are defined as peaks in the expression strength exceeding $R_{\text{THRES}}=5$.

We designed a 1-day CPP task in which mice form a mnemonic association between a spatial environment and rewards (sucrose). We implanted mice with dCA1 tetrodes to perform multichannel extracellular recordings of the activity of PYRs throughout all behavioral sessions of this 1-day protocol (Figure 2A). Each day started with a pre-test session to establish the spontaneous preference of each mouse for one of the two distinct and novel enclosures connected by a bridge (Figures 2B and 2C). The bridge was next removed in conditioning sessions during which each mouse first explored its non-preferred enclosure baited with drops of a sucrose solution (+Suc.; Figure 2B), and then explored its preferred enclosure baited with an equal volume of water drops (+Wat.; Figure 2B). One hour after the last conditioning session, the bridge was reinserted and mice were re-exposed to the apparatus during a test session to assess CPP memory (Figures 2B and 2C). We detected dCA1 assembly patterns formed by groups of co-active PYRs (25-ms time bins) during exploration of the CPP apparatus prior to the test (Figures 2D and S2). These dCA1 firing patterns were spatially tuned and thus provide a spatial representation of that apparatus (Figure 2D). During the test, mice spent more time in the sucrose-conditioned enclosure, indicating successful CPP memory (Figure 2E); this spatial behavior coincided with the reinstatement of dCA1 assemblies (Figures 2F and 2G).

**Figure S3:**
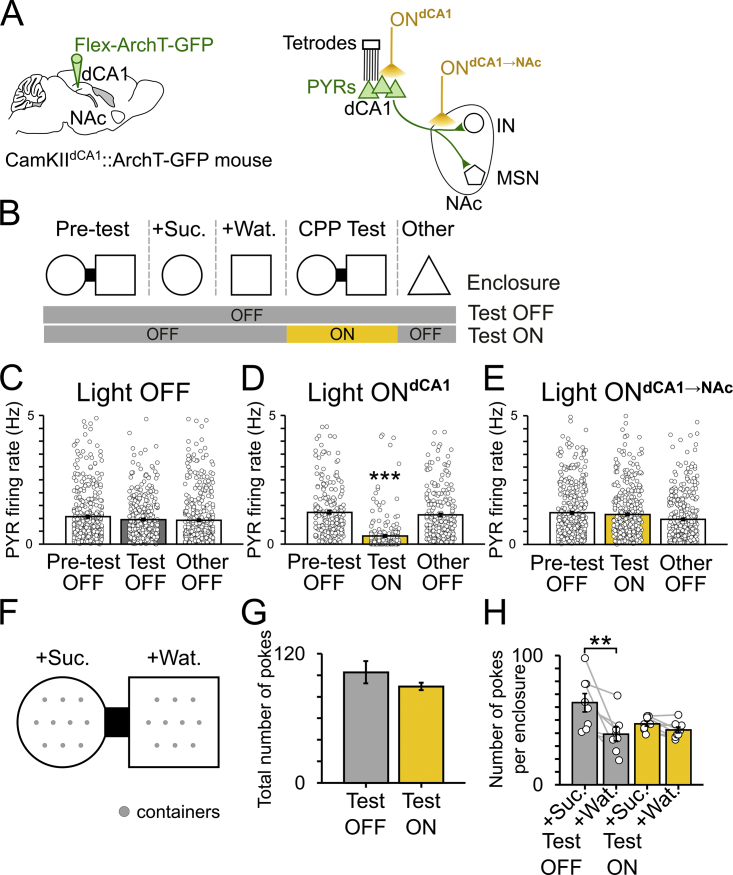
Preserved dCA1 PYR and Reward-Seeking Activities during Axon-Directed Light Delivery in the NAc, Related to Figure 2 (A) dCA1 PYRs of CamKII-Cre mice were transduced with ArchT-GFP and their firing activity monitored using tetrodes. dCA1 somata-targeting (ON^dCA1^) or axon-targeting (ON^dCA1→NAc^) light delivery was performed using optic fibers inserted above either dCA1 or NAc, respectively. (B) Mice performed each day a one-day CPP and explored an additional enclosure (“Other”). Yellow light pulses were delivered during CPP test (ON^dCA1^: one 10min-pulse; ON^dCA1→NAc^: 20x30sec-pulses over 15min). (C–E) Firing rate (bar charts: mean ± SEM) of dCA1 PYRs recorded throughout the Pre-test, CPP Test and “Other” sessions (C: n = 493 light OFF PYRs; D: 227 ON^dCA1^ PYRs; E: 456 ON^dCA1→NAc^ PYRs). Each dot represents a single neuron. ^∗∗∗^p < 0.001 compared to Pre-test and Other, 1-way ANOVA with Tukey multiple comparisons of means. (F–H) Reward-seeking behavior during CPP test. During the conditioning session (B), each enclosure contained ten small plastic containers (F), each filled with either a drop of sucrose (+Suc.) or water (+Wat.). Active reward search was quantified by the number of pokes to the (no longer baited) containers during CPP test in both light OFF and ON^dCA1→NAc^ days. Note that light ON delivery during CPP test did not affect the average total number of pokes was similar between light conditions (G; mean ± SEM; p = 0.192, paired t test), thus ruling out a light-induced motivational deficit. However, light ON delivery prevented the spatially orientated poke behavior toward the sucrose-paired enclosure seen in light OFF (H; ^∗∗^p < 0.01, ANOVA with Tukey multiple comparisons of means), in line with an impaired CPP behavior (see Figure 2K). Abbreviations: dorsal hippocampal CA1 (dCA1), nucleus accumbens (NAc), pyramidal cells (PYRs), medium-sized spiny neuron (MSN), interneuron (IN), conditioned place preference (CPP).

We assessed whether behavioral performance in this task is dependent on dCA1 neuronal activity. We transduced dCA1 PYRs with ArchT-GFP in additional CamKII-Cre mice (Figure 2A). We inserted optic fibers bilaterally above the dCA1 of these CamKII^dCA1^::ArchT-GFP mice for optogenetic silencing of PYRs during CPP test (light ON^dCA1^); concurrent implantation of dCA1 tetrodes allowed for simultaneous monitoring of PYR ensembles (Figure 2A). The somata-targeting delivery of yellow light during the test not only suppressed dCA1 PYR firing (Figures S3A–S3D) but also prevented CPP behavior (Figure 2H) and the reinstatement of dCA1 CPP assemblies (Figures 2I and 2J). The behavioral manifestation of CPP memory thus requires the unhindered reinstatement of dCA1 representations.

### CPP Further Requires a Functional dCA1→NAc Pathway

Next, we tested the contribution of the dCA1→NAc projection to CPP memory. We bilaterally implanted another group of CamKII^dCA1^::ArchT-GFP mice with optic fibers above the NAc; concurrent implantation of dCA1 tetrodes allowed for simultaneous monitoring of PYR ensembles (Figure 2A). Optogenetic disruption of dCA1→NAc pathway was obtained by delivering yellow light to dCA1 ArchT-containing PYR axon terminals in the NAc during CPP test (light ON^dCA1→NAc^). This intervention prevented the behavioral expression of CPP memory (Figure 2K), an impairment similar to that resulting from the direct silencing of dCA1 PYRs (light ON^dCA1^; Figure 2H). However, reward-seeking behavior was unaltered when disrupting the dCA1→NAc pathway (Figure S3F–S3H). Both dCA1 assembly reinstatement (Figures 2L and 2M) and PYR firing rate (Figure S3E) were also preserved. Together, these results show that the dCA1 representation of the CPP apparatus is necessary, but not sufficient, to act on appetitive memory retrieval; a functional dCA1→NAc pathway is yet required for the behavioral manifestation of the CPP memory upon its neuronal reinstatement.

### dSub Inputs to NAc and dCA1 Inputs to dSub Are Dispensable for CPP

We evaluated the extent to which the role of the neuronal pathway linking dorsal hippocampus to NAc in CPP memory retrieval was selective to the direct dCA1 inputs. In addition to the CA1 region of the dorsal hippocampal formation, the NAc receives excitatory inputs from the dorsal subiculum (dSub), which is also a major recipient of dCA1 PYR projections and an important circuit for memory (e.g., Cembrowski et al., 2018, Groenewegen et al., 1987, Morris et al., 1990, Roy et al., 2017, van Strien et al., 2009). Therefore, it is possible that dCA1 PYRs also support CPP by using dSub as an intermediate circuit.

**Figure 3:**
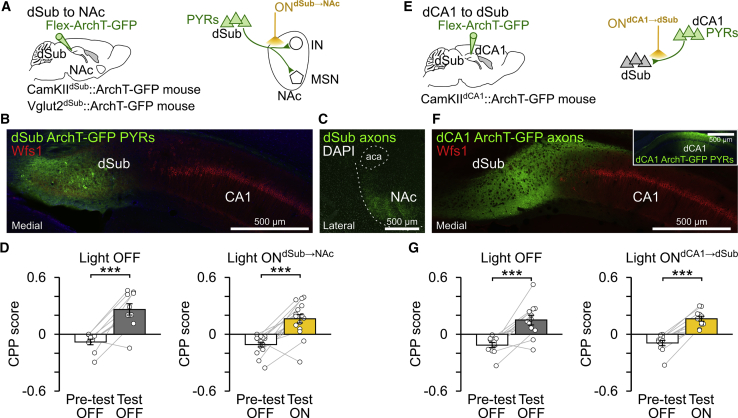
CPP Memory Does Not Require dSub Inputs to NAc or dCA1 Inputs to dSub (A) Dorsal subiculum (dSub) PYRs were transduced with ArchT-GFP, and yellow light was delivered on ArchT-expressing axons in NAc (ON^dSub→NAc^) using optic fibers inserted bilaterally above the NAc of dSub-transduced (CamKII-Cre or Vglut2-Cre) mice. Each day, mice performed (n = 26 days from six mice) the 1-day CPP task (like in Figure 2B). (B and C) ArchT-GFP expression in dSub cell bodies (B) and their axons in NAc (C). Wfs1 staining shows caudal portion of hippocampal CA1 adjacent to dSub. (D) CPP score (mean ± SEM) during pre-test and test (n = 10 light OFF; 16 ON^dSub→NAc^ days). Light was delivered during CPP ON^dSub→NAc^ test (one 10-min pulse; like in Figure 2K). (E) dCA1 PYRs were transduced with ArchT-GFP, and yellow light was delivered on their axons in dSub (ON^dCA1→dSub^) using optic fibers bilaterally inserted above dSub. Mice performed each day (n = 23 days from 6 mice) the 1-day CPP task. (F) dCA1 ArchT-expressing axons in dSub. The inset confocal image shows dCA1 transduced with ArchT-GFP. (G) CPP score (mean ± SEM) during pre-test and test (n = 12 light OFF; 11 ON^dCA1→Sub^ days). Light was delivered during CPP test (one 10-min pulse over 15 min; like in Figure 2K). ^∗∗∗^p < 0.001, paired t tests. See also Figure S4.

Accordingly, we examined the contribution of the dSub→NAc pathway to CPP. In additional mice, we transduced dSub PYRs with ArchT-GFP (Figures 3A and 3B) and bilaterally inserted optic fibers above the NAc for yellow light delivery to dSub ArchT-GFP axons (Figure 3C) during CPP test. Mice were subjected to the same experimental paradigm used to inactivate dCA1 axons in NAc (light ON^dCA1→NAc^); this time applied to dSub axons (light ON^dSub→NAc^). While the optogenetic inactivation of dCA1 axon terminals in NAc prevented CPP (Figure 2K), disrupting the dSub→NAc pathway preserved it (Figure 3D). This shows a dissociation between two projections from the dorsal hippocampal formation to the NAc in spatial appetitive memory.

**Figure S4:**
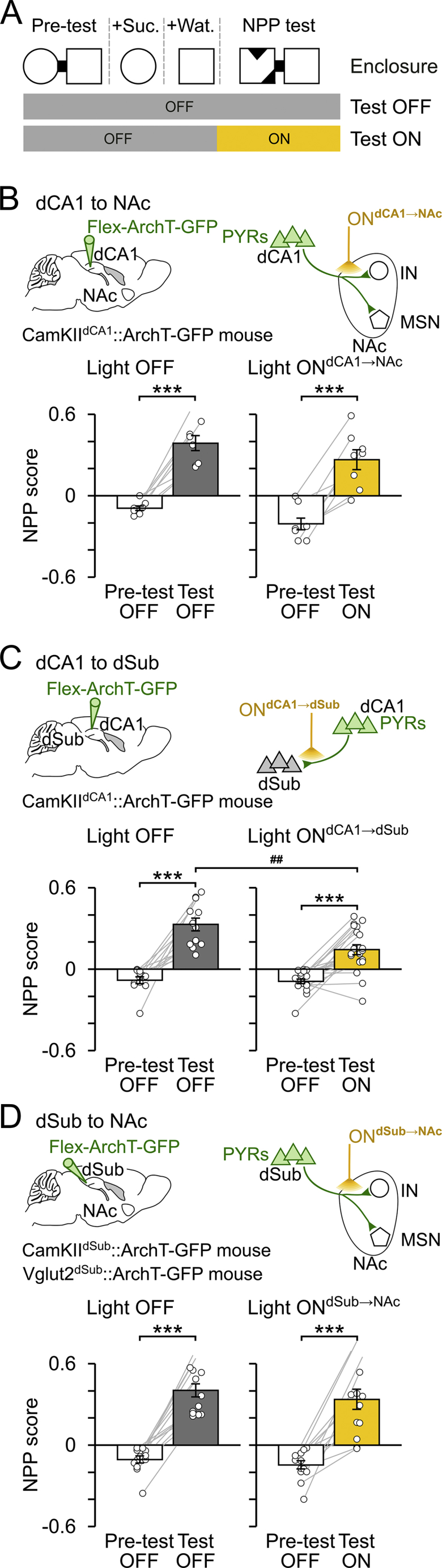
Effect of Axon-Directed Optogenetic Inactivation of dCA1 or dSub Inputs during Spatial Novelty Detection, Related to Figures 2K and 3 (A) Mice were tested for their ability to detect spatial novelty using a one-day novel place preference (NPP) task during which the sucrose-paired enclosure of the conditioning sessions was replaced by a novel environment in the test session. In light ON days, yellow light (one 10min-pulse) was delivered during test. The NPP score (mean ± SEM) was measured during test as the time spent in the novel enclosure minus that in the other enclosure over the sum, and similarly in the pre-test session. Positive NPP scores indicate successful spatial novelty detection. (B) dCA1 PYRs were transduced with ArchT-GFP and yellow light was delivered onto their axons in NAc (light ON^dCA1→NAc^) using optic fibers inserted bilaterally above the NAc of dCA1-transduced CamKII-Cre mice (n = 16 total NPP days; n = 8 light OFF and 8 light ON^dCA1→NAc^). ^∗∗∗^p < 0.001, paired t tests. (C) dCA1 PYRs were transduced with ArchT-GFP and yellow light was delivered onto their axons in dSub (light ON^dCA1→dSub^) using optic fibers inserted bilaterally above the dSub of dCA1-transduced CamKII-Cre mice (n = 32 total NPP days from 6 mice; n = 12 light OFF and 20 light ON^dCA1→dSub^). ^∗∗∗^p < 0.001, paired t tests. Note the reduced NPP scores caused by optogenetic disruption of dCA1 PYR projection to dSub (^##^ p = 0.0011, ANOVA with Tukey multiple comparisons of means). (D) dSub PYRs were transduced with ArchT-GFP and yellow light was delivered on their axons in NAc (light ON^dSub→NAc^) using optic fibers inserted bilaterally above the NAc of dSub-transduced (CamKII-Cre or Vglut2-Cre) mice (n = 26 total NPP days from 6 mice; n = 13 light OFF and 13 light ON^dSub→NAc^). ^∗∗∗^p < 0.001, paired t tests.

We further tested whether the role of dCA1 inputs in CPP was selective to their NAc recipient or whether it is supported by any circuit outflow of dCA1, such as dSub. We used additional CamKII^dCA1^::ArchT-GFP mice in which inserting optic fibers bilaterally above dSub allowed us to inactivate dCA1 axons in dSub during CPP test (light ON^dCA1→dSub^; Figures 3E and 3F). Whereas mice under light ON^dCA1→NAc^ displayed impaired CPP memory (Figure 2K), mice with blocked dCA1→dSub pathway (light ON^dCA1→dSub^) exhibited intact CPP (Figure 3G). This shows that the dCA1→dSub pathway is dispensable for the behavioral expression of spatial appetitive memory. However, we observed that dCA1 inputs to dSub participate in successful (unconditioned) spatial novelty detection, as tested in a novel place preference task (Figure S4).

These findings demonstrate that CPP memory retrieval engages direct dCA1 inputs to NAc, while indirect pathways involving dSub are dispensable.

### Disruption of dCA1→NAc Pathway Affects a Subset of NAc FSIs

**Figure 4:**
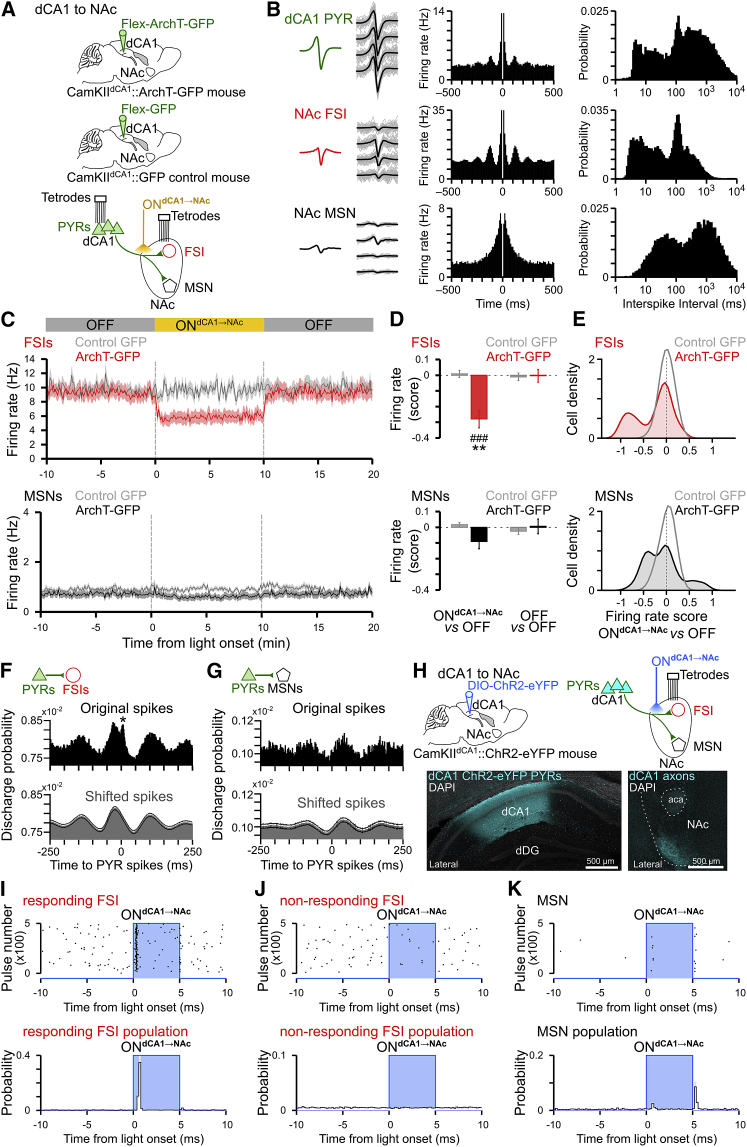
dCA1 Inputs Drive NAc MSNs and of a Subset of FSIs (A) dCA1 PYRs were transduced with either the ArchT-GFP or the control GFP-only construct. Dual-site tetrode implant allowed for simultaneous monitoring of dCA1 and NAc ensembles (n = 671 PYRs and 271 NAc neurons, including 169 MSNs and 65 FSIs from five behaving CamKII^dCA1^::ArchT-GFP mice; n = 181 NAc neurons, including 107 MSNs and 35 FSIs from two behaving CamKII^dCA1^::GFP control mice). Optic fibers inserted above NAc allowed delivering yellow light to dCA1 axons in NAc (ON^dCA1→NAc^) in both CamKII^dCA1^::ArchT-GFP (“ArchT-GFP”) and CamKII^dCA1^::GFP (“control-GFP”) mice. (B) Examples of a recorded PYR, FSI, and MSN. From left to right shown for each neuron the average spike waveform, example spikes recorded on each channel of corresponding tetrode, spike train auto-correlogram, and inter-spike interval distribution. See also Figure S5. (C) Firing rate of FSIs and MSNs (mean ± SEM; 50-ms bins) relative to light ON^dCA1→NAc^ delivery (one 10-min pulse) and recorded from the NAc of CamKII^dCA1^::GFP control mice (in gray) or CamKII^dCA1^::ArchT-GFP mice (with MSNs in black; FSIs in red). See Figure S5I for the distinct firing responses of light ON^dCA1→NAc^ responding and non-responding FSIs separately. (D and E) Corresponding firing rate changes (scores) of FSIs and MSNs (D: mean ± SEM; E: distributions of rate scores using a kernel density approach) (Silverman, 1981). For each cell, light-driven score corresponds to the firing rate in light ON minus that in light OFF divided by the sum; a firing rate score was also computed between the two OFF epochs either side of the ON^dCA1→NAc^ (as shown in C) for comparison. Note that light ON^dCA1→NAc^ delivery to dCA1 axons in NAc of CamKII^dCA1^::ArchT-GFP mice caused bidirectional rate changes within the MSN population (with some MSNs showing increased firing rate, while others decreased firing). In the same time, light delivery to dCA1 ArchT-GFP axons drastically decreased the rate of a subset of FSIs (i.e., FSIs with a score toward –1). The activity of the other FSIs remained unaffected (i.e., other scores near 0). All FSIs recorded from CamKII^dCA1^::ArchT-GFP mice pooled together in Figure 4C (see also Figure S5I). The same light delivery did not cause rate changes of FSIs (E; top gray curve) and MSNs (E, bottom gray curve) in CamKII^dCA1^::GFP control mice (i.e., all scores near 0). ^∗∗^p < 0.01 unpaired t test FSIs versus corresponding MSNs just below, ^###^ p < 0.001 compared to zero. (F and G) Temporal relationship between spike discharge of simultaneously recorded dCA1 and NAc neurons. Time zero equals spike times of PYRs, used as reference. Black histograms: population cross-correlograms computed from the original spike trains of PYR-FSI (F) and PYR-MSN (G) cell pairs. Note the theta-paced spiking of both PYR-FSI and PYR-MSN pairs, with phase opposition. Gray histograms: control cross-correlograms computed from PYR spikes shifted to random theta cycles with preserved (original) theta phase. Note the sharp, short-latency increase of FSIs spike probability following the original, but not the shifted, PYRs discharge (black asterisk; PYR-to-FSI offset: 3.2–8 ms), suggestive of a direct entrainment of some NAc FSIs by dCA1 PYRs. (H) dCA1 PYRs of CamKII-Cre mice were transduced with ChR2-eYFP. Tetrodes were implanted for NAc ensemble recordings (n = 222 NAc neurons including 128 MSNs and a total of 64 FSIs from two behaving CamKII^dCA1^::ChR2-eYFP mice). Optic fibers inserted above NAc allowed delivering blue light to photo-stimulate dCA1 axons (ON^dCA1→NAc^, 5-ms pulses). (I–K) Spike discharge of NAc FSIs and MSNs in response to *in vivo* photo-stimulation of dCA1 ChR2-eYFP axons in NAc of CamKII^dCA1^::ChR2-eYFP mice (H). Top row: examples of single-neuron raster plots for a dCA1-responding FSI (I), a non-responding FSI (J) and a responding MSN (K). Note the short-latency light-driven spiking of the responding FSI compared to the absence of firing response of the other FSI (both FSIs simultaneously recorded from the same mouse, same tetrode). Note also the longer-latency spiking of the MSN. Bottom row: corresponding spike discharge probabilities (mean ± SEM; 0.2-ms bins) for the populations of dCA1-responding FSIs (n = 12), non-responding FSIs (n = 52), and MSNs (n = 128), relative to the light ON^dCA1→NAc^ onset.

**Figure S5:**
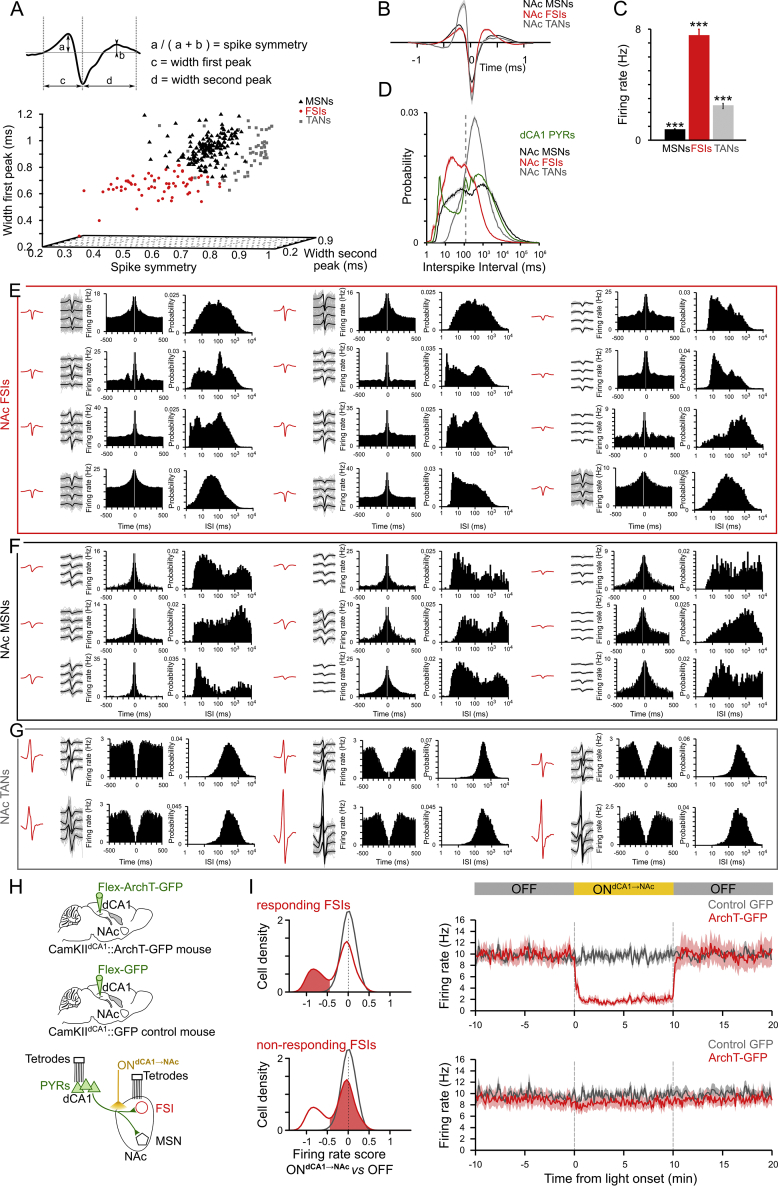
Physiological Identification of NAc Neuronal Types, Related to Figure 4 (A) The classification of tetrode-recorded NAc neurons was based on spike waveform parameters. Top: the average waveform of extracellular spikes was described using three parameters: the duration of the first positive component (“first peak”), the duration of the second positive component and the relative amplitude of the two (spike symmetry). Bottom: the three parameters plotted in the three-dimensional parameter space. The three clusters identifying NAc neurons as FSIs, MSNs or TANs are color-coded. (B and C) The average spike waveforms (±SEM shaded area; B) and the firing rate during spatial exploration (mean ± SEM; C) of the neurons from each cluster. Note that the identified FSIs exhibited a brief spike duration and discharged action potentials at substantially higher rates compared to identified MSNs and TANs, in line with previous studies (e.g., Berke et al., 2004). In contrast, identified MSNs exhibited the lowest firing rate. ^∗∗∗^p < 0.001, 1-way ANOVA with Tukey multiple comparisons of means. (D) Distributions of inter-spike intervals (ISI; mean ± SEM; 1-ms bin) of identified FSIs, MSNs and TANs. For comparison, the ISI distribution of recorded dCA1 pyramidal cells (PYRs) is shown. Note the sharp increase in ISI probability at ∼100ms (theta cycle duration) for NAc FSIs and dCA1 PYRs (vertical gray dashed line). (E–G) Examples of identified NAc FSIs, MSNs and TANs. From left to right, the average spike waveform, example spikes recorded on each channel of corresponding tetrode, spike train auto-correlogram and inter-spike interval distribution are shown, for each neuron. (H) dCA1 PYRs of CamKII-Cre mice were transduced with either ArchT-GFP or the GFP-only control construct. NAc neurons were recorded from both CamKII^dCA1^::ArchT-GFP (“ArchT-GFP”) and CamKII^dCA1^::GFP (“Control-GFP”) mice. Optic fibers inserted above the NAc allowed for yellow light delivery targeting dCA1 axons in the NAc of both mouse groups. (I) The firing rate of NAc FSIs recorded from CamKII^dCA1^::ArchT-GFP mice (red) and CamKII^dCA1^::GFP control mice (gray) is shown relative to the light ON^dCA1→NAc^ delivery. For this analysis, the FSIs recorded from CamKII^dCA1^::ArchT-GFP mice were split according to their firing change caused by light delivery shown in Figure 4E. *Top left panel*: distribution of scores with red shaded area depicting FSIs with light ON^dCA1→NAc^ suppressed firing. *Bottom left panel*: distribution of scores with red shaded area depicting simultaneously recorded FSIs with light-unchanged firing rate. The FSIs recorded from CamKII^dCA1^::GFP control mice (in gray) are also shown for comparison. Note the significant firing suppression of the light-responding FSIs compared to both the non-responding FSIs (simultaneously recorded from CamKII^dCA1^::ArchT-GFP mice) and the FSIs recorded from CamKII^dCA1^::GFP control mice (right panels; mean ± SEM; 50ms bins). Light delivery did not cause rate changes in CamKII^dCA1^::GFP control mice (i.e., all scores near 0). See Figure 4C for all FSIs recorded from CamKII^dCA1^::ArchT-GFP mice pooled together. We further noted a difference in the depth of modulation by dCA1 theta oscillations between the two subsets of NAc FSIs recorded from CamKII^dCA1^::ArchT-GFP mice (Rayleigh vector length in light OFF: light ON^dCA1→NAc^ responding FSIs = 0.115 ± 0.043, non-responding FSIs = 0.035 ± 0.006, p = 0.0352, unpaired t test). Abbreviations: dorsal hippocampal CA1 (dCA1), nucleus accumbens (NAc), pyramidal cells (PYRs), medium-sized spiny neuron (MSNs), fast-spiking interneurons (FSIs), tonically active neurons (TANs), inter-spike intervals (ISI).

We observed that dCA1 PYRs establish synaptic contacts with both MSNs and putative interneurons in the NAc. We thus examined *in vivo* whether dCA1 PYR spiking indeed influences these two cell populations. We simultaneously recorded dCA1 and NAc neurons in behaving CamKII^dCA1^::ArchT-GFP mice combined with optogenetic inactivation of the dCA1→NAc pathway (light ON^dCA1→NAc^; Figure 4A). To rule out possible effects of light delivery per se, we also recorded the NAc of CamKII-Cre mice with dCA1 PYRs transduced by a viral construct only coding for GFP (CamKII^dCA1^::GFP control mice; Figure 4A). We identified putative NAc MSNs and FSIs based on their spike waveform features (Figures 4B, S5A, and S5B). We noted that the spike discharge of certain FSIs (Figures 4B and S5E) is paced at theta frequency (4–12Hz; ∼125-ms-long cycles) (Berke et al., 2004). Because this rhythm dominates dCA1 activity during spatial behavior (Buzsáki, 2010), we reasoned that the theta-rhythmic profile of these NAc interneurons might reflect their direct innervation by dCA1 PYRs. Consistent with this prediction, inactivating the dCA1→NAc pathway in CamKII^dCA1^::ArchT-GFP mice decreased the average firing rate of the NAc FSI population (Figures 4C and 4D; *ArchT-GFP*). Of note, this effect was accounted for by the strong suppression of some, but not all, FSIs, as shown in the distribution of single-neuron light-driven rate changes (Figures 4E, *ArchT-GFP*, and Figures S5H and S5I). The light ON^dCA1→NAc^ delivery did not significantly affect the overall MSN firing in CamKII^dCA1^::ArchT-GFP mice (Figures 4C and 4D, *ArchT-GFP*), but caused bidirectional rate changes within this population (Figure 4E, *ArchT-GFP*). Importantly, none of the MSNs nor the FSIs recorded from CamKII^dCA1^::GFP control mice exhibited light-altered firing (Figure 4C–4E, *control GFP*).

To investigate further the temporal relationship between dCA1 and NAc spiking, we calculated the discharge probability of both MSNs and FSIs relative to PYR spike times (in light OFF). Remarkably, FSIs (Figure 4F), but not MSNs (Figure 4G), exhibited a sharp and short latency response to PYR spiking (*asterisk*). This was not a mere product of their theta phase modulation (Figures 4F and 4G; see temporally shifted spike controls). We also noticed that the population discharge of both MSNs and FSIs oscillates at theta frequency, with phase opposition (Figures 4F and 4G).

Collectively, these data supported the hypothesis that dCA1 PYR inputs influence both MSNs and FSIs, albeit a more prominent effect on the latter. We examined this by directly stimulating dCA1 axons transduced with the blue light (473 nm)-driven neural activator channelrhodopsin-2 (ChR2)-eYFP, while recording NAc ensembles in CamKII^dCA1^::ChR2-eYFP mice (Figure 4H). Activating dCA1 ChR2-eYFP axons in the NAc entrained at short latency the spike discharge of a subset of FSIs (Figures 4I and 4J), in line with our findings above (Figures 4E and S5I). Stimulating dCA1 axons in the NAc also drove MSN spiking (Figure 4K), although this occurred after dCA1-responding FSI spiking (Figure 4I). These results demonstrate that dCA1 inputs can entrain MSNs and suggest that dCA1 PYRs shape NAc firing output by further recruiting a subset of FSIs.

### dCA1 PYRs Form Specialized Synaptic Contacts with NAc PV Interneurons

**Figure 5:**
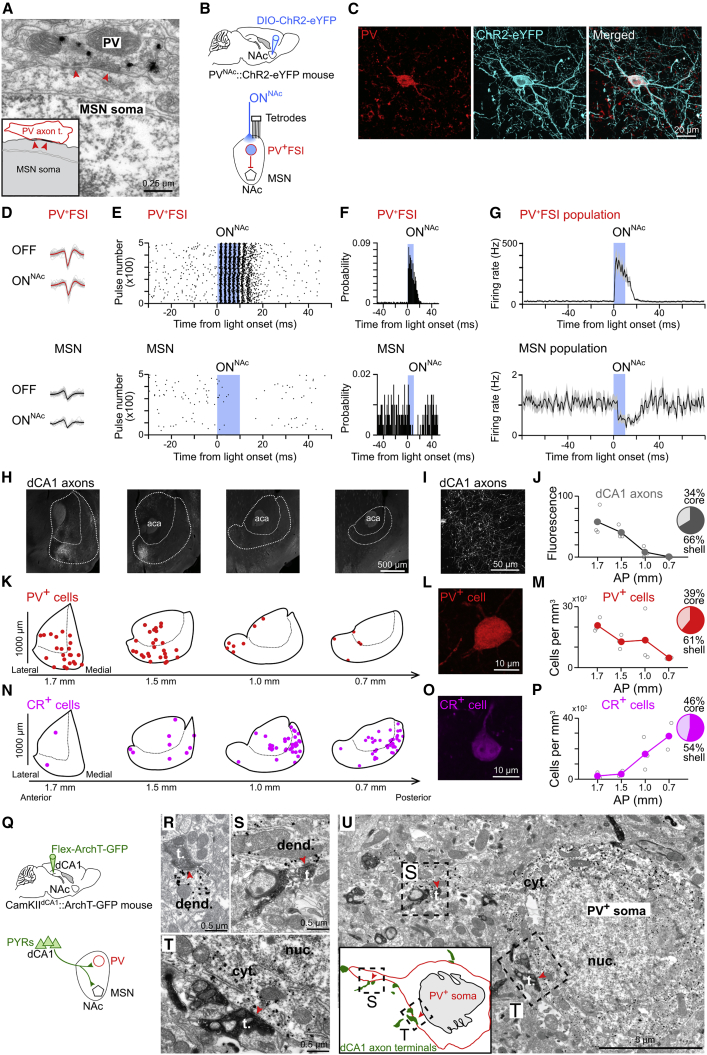
NAc PV^+^FSIs Receive Monosynaptic Inputs from dCA1 PYRs (A) PV^+^ axon terminal (immunogold-labeled) forming a symmetric synapse (red arrowheads) with the soma of an MSN. (B and C) NAc of PV-Cre mice was injected with a ChR2-eYFP viral construct (B) to transduce PV^+^ interneurons (C). Tetrodes were subsequently implanted in the NAc and optic fibers above for ensemble recording and blue light delivery (ON^NAc^, 10-ms pulses; four mice), respectively. (D–F) Examples of one opto-tagged NAc PV^+^FSI (top row) and one MSN (bottom row) recorded *in vivo*. From left to right, shown are (D) example spikes (gray traces) with superimposed mean waveforms (red/black traces) in light OFF and ON^NAc^ conditions, (E) raster plots showing the short-latency light-driven spiking of the FSI and the decreased spiking of the MSN, and (F) their spike discharge probabilities. The sharp peak at short latency identifies this FSI as an opto-tagged PV^+^ cell. (G) Firing rate of opto-tagged PV^+^FSIs (n = 8) and MSNs (n = 56) relative to the light onset (mean ± SEM; 1ms bins). Note that the light-driven firing of PV^+^FSIs was associated with decreased average firing of the MSN population. (H–P) Distribution of (H–J) dCA1 ArchT-GFP axons, (K–M) PV^+^ interneurons and (N–P) calretinin-expressing (CR^+^) interneurons along the rostro-caudal axis and the core versus shell regions of the NAc. (K and N) Each dot represents a single immuno-positive neuron in the optical slice. Confocal images showing (I) dCA1 axons, (L) a PV^+^ interneuron and (O) a CR^+^ interneuron in the NAc. Fluorescence intensity of ArchT-GFP dCA1 axons (J) and densities of PV^+^ (M) and CR^+^ (P) cells (three mice). Numbers associated with each pie chart represent the percentages of axons (J) or cell bodies (M and P) in NAc core and shell. See also Figures S6A–S6F. (Q) dCA1 PYRs of CamKII-Cre mice were transduced with ArchT-GFP. (R–U) GFP-containing dCA1 PYR axon terminals (labeled with DAB) forming synaptic contacts (red arrowheads) with a distal dendrite (R), a proximal dendrite (S), and a soma (T) of NAc PV^+^ cells (U; labeled by immunogold particles). See also Figures S6G–S6I. PV, parvalbumin; CR, calretinin; t., axon terminal; dend., dendrite; cyt., cytoplasm; nuc., nucleus.

**Figure S6:**
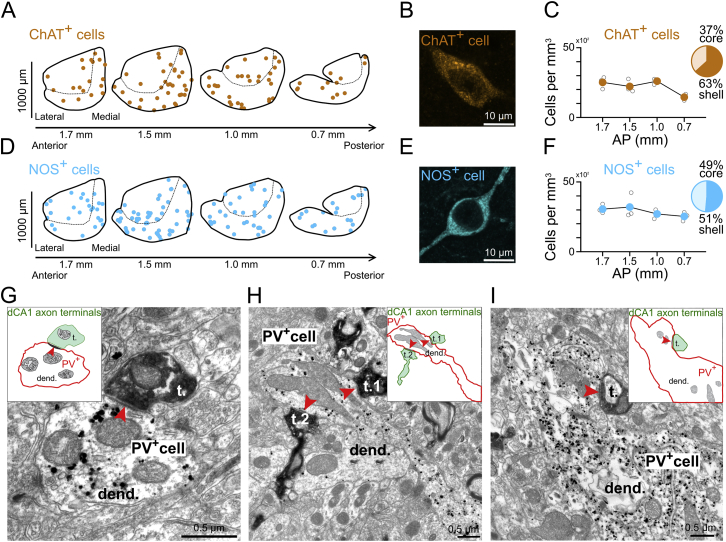
dCA1 PYRs Are Monosynaptically Connected with NAc PV^+^ Interneurons, Related to Figure 5 (A–F) Distribution of interneurons expressing ChAT (A–C) or NOS (D–F) along the rostro-caudal axis and the core versus shell regions of the NAc. Each dot represents a single ChAT or NOS immuno-positive neuron (A, D). High magnification confocal images showing a ChAT cell (B) and a NOS cell (E) in the NAc. Density of ChAT (C) and NOS (F) cells (n = 3 mice). Note that ChAT- and NOS- expressing interneurons are evenly distributed along the NAc rostro-caudal axis. The percentages associated with each pie chart represent the percentages of interneurons in NAc core and shell. (G–I) Electron micrographs showing GFP-containing dCA1 axon terminals (labeled with DAB) making synaptic contacts (red arrowheads) with NAc PV interneurons (labeled by immunogold particles). Note that dCA1 terminals target both distal (G and H) and proximal (I) dendrites of PV cells. Note also that an individual PV dendrite can form multiple synapses with dCA1 axons in a single plane (H). Schematic of synapses shown as insets. Abbreviations: PV: parvalbumin; ChAT: choline acetyltransferase; NOS: neuronal isoform of nitric oxide synthase; t.: axon terminal; dend.: dendrite.

GABAergic interneurons expressing the calcium-binding protein parvalbumin (PV) represent prime candidates to mediate the influence of dCA1 on NAc output (Tepper et al., 2010). Indeed, we observed that PV^+^ axons formed symmetric synapses (Gray’s type II; typical of those formed by GABAergic axons) with the somata and dendrites of MSNs (Figure 5A). We confirmed that opto-tagged PV^+^ interneurons, recorded from awake mice expressing ChR2-eYFP in NAc PV^+^ cells (light ON^NAc^ in PV^NAc^::ChR2-eYFP mice; Figures 5B and 5C), exhibit a fast-spiking phenotype (Figures 5B–5G), and that their photo-stimulation decreased MSN population firing (Figures 5D–5G). We also found that the distribution of both dCA1 PYR axons and PV^+^ somata overlapped, being both biased to the rostral part of the NAc (Figures 5H–5M). In contrast, interneurons expressing the calcium-binding protein calretinin exhibited a caudal distributional bias, to NAc regions where dCA1 innervation was sparse (Figures 5N–5P). Other interneuron types were distributed evenly across the NAc rostro-caudal axis (Figures S6A–S6F). Finally, we also found that dCA1 axon terminals establish asymmetric synapses with the aspiny dendrites of NAc PV^+^ interneurons, at both distal dendritic sites (Figures 5Q, 5R, S6G, and S6H) and proximal sites (Figures 5S, 5U, and S6I), as well as with their somata (Figures 5T and 5U). We did not observe this triple targeting profile for MSNs, as only their distal dendrites receive dCA1 inputs. This specialized innervation of NAc PV interneurons, combined with the time-locked response of FSIs to dCA1 inputs (Figure 4), suggest that dCA1 PYRs drastically influence MSN firing output using local PV^+^FSIs.

### dCA1 PYRs Exert Feedforward Inhibition onto Postsynaptic MSN Targets

**Figure 6:**
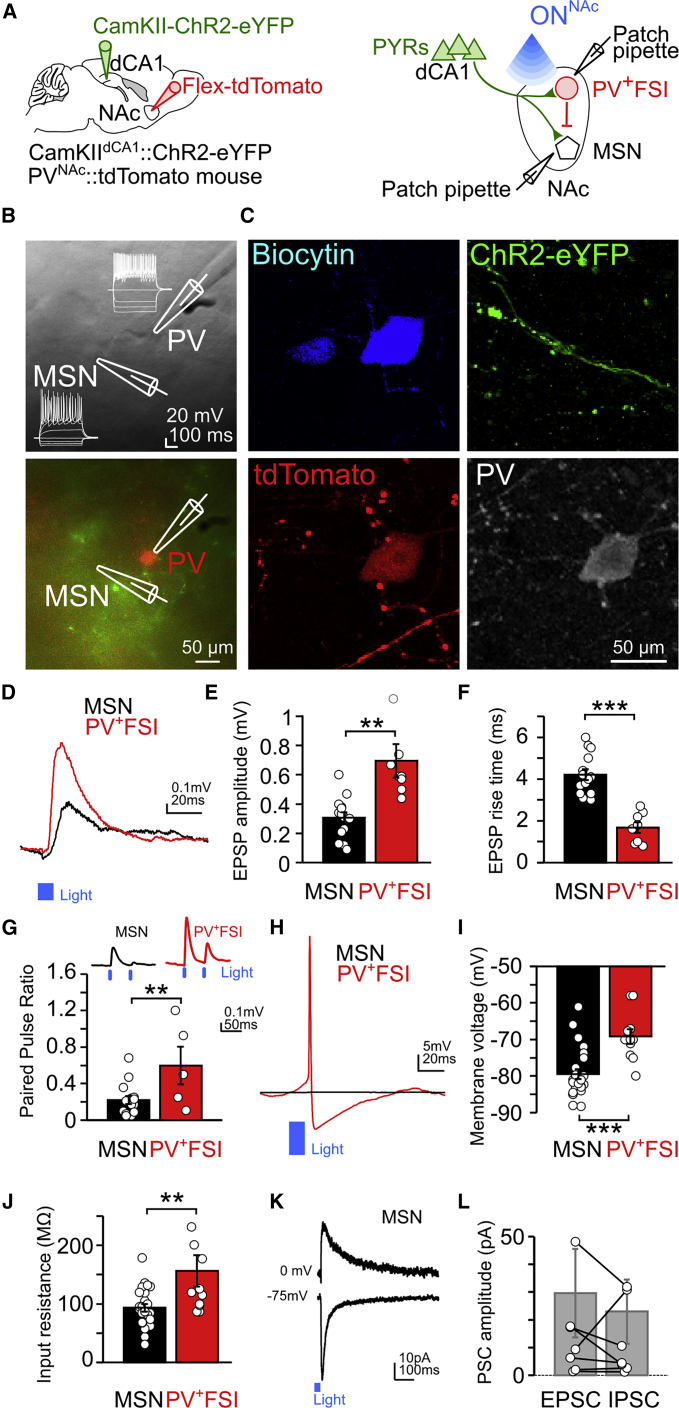
dCA1 Axons Drive Both MSN and PV^+^FSI Postsynaptic Responses and Exert Feedforward Inhibition on dCA1-Connected MSNs (A) dCA1 PYRs were transduced with CamKII-ChR2-eYFP and NAc PV^+^ interneurons were visualized by the tdTomato fluorescent reporter in PV-Cre mice. Whole-cell patch-clamp recordings of NAc FSIs and MSNs were performed in combination with blue light wide-field NAc optogenetic stimulation of dCA1 ChR2-eYFP axons (2- to 3-ms duration light ON^NAc^ pulses). (B) Whole-cell patch-clamp configuration. Top: Dodt contrast image of simultaneously recorded NAc FSI and MSN and corresponding current-voltage plots highlighting the responses to depolarizing current steps. Bottom: corresponding fluorescence image with simultaneously recorded tdTomato-expressing PV^+^FSI and unlabeled MSN. (C) The neurons recorded in (B) and labeled with biocytin (top left), with the presence of dCA1 ChR2-eYFP axons (top right). The tdTomato-expressing FSI (bottom left) is PV-immunopositive (bottom right). (D) Example EPSPs evoked in an MSN and PV^+^FSI in response to optogenetic stimulation of dCA1 axons. Note the larger EPSP in the PV^+^FSI. (E and F) Optogenetic stimulation of dCA1 axons evoked significantly larger EPSPs (E) in PV^+^FSIs with shorter rise times (F). One off-display PV^+^FSI data point (E, 1.46 mV). A total of 8 of 16 PV cells and 13 of 25 MSNs exhibited an EPSP response to activation of dCA1 axons. None of the neighboring PV-negative interneurons responded to photo-stimulation of dCA1 inputs (n = 6). (G) PV^+^FSIs exhibited less paired-pulse depression than MSNs following successive photo-stimulations of dCA1 axons (paired-pulse ratio: n = 5 PV^+^FSIs and 15 MSNs; with EPSP amplitude: PV^+^FSIs = 0.79 ± 0.18mV, MSN = 0.22 ± 0.04 mV, p = 0.01, Wilcoxon test, n = 5 cell-pair recordings, where both PV^+^FSIs and MSN cells received dCA1 inputs). Example traces for one MSN and one PV^+^FSI recorded during paired-pulse stimulations of dCA1 axons shown on top. (H) Stimulation of dCA1 axons with increased light intensity resulted in action potential discharges by a PV^+^ FSI, but not by a simultaneously recorded MSN. (I) Resting membrane voltage for recorded PV^+^FSIs and MSNs. PV^+^FSIs were significantly more depolarized than MSNs. (J) Input resistance was significantly higher in PV^+^FSIs compared to MSNs. We did not observe significant differences in the electrophysiological properties of FSIs and MSNs that did or did not respond to dCA1 axon stimulation (Table S1). (K and L) dCA1 axon stimulation elicited both EPSCs and IPSCs in dCA1-connected MSNs. Voltage-clamp recordings were performed for MSNs that showed dCA1-evoked EPSPs under current-clamp (n = 8 dCA1-responding MSNs of 18 tested MSNs; all dCA1-responding MSNs exhibited both EPSCs and IPSCs; E/I ratio = 1.58 ± 0.45). IPSCs and EPSCs recorded by holding MSNs at 0 mV and −75 mV, respectively. Shown in (K) are average IPSC (top) and EPSC (bottom) traces for one MSN (20 sweeps of 473nm dCA1 axon stimulation). Peak amplitude of recorded EPSCs and IPSCs shown in (L) (with off-display pair of data points for one MSN: EPSC = 134.80 pA, IPSC = 98.04 pA). Note that all recorded dCA1-responding MSNs received both excitatory and inhibitory currents upon dCA1 input stimulation. ^∗∗^p < 0.01, ^∗∗∗^p < 0.001, Mann-Whitney U test. See also Table S1.

To test the functional difference of dCA1 inputs onto MSNs and PV^+^FSIs, we targeted dCA1 PYRs of PV-Cre mice with a CamKII-ChR2-eYFP viral vector and NAc PV cells with the expression of the tdTomato reporter (Figure 6A). We observed dCA1 axons (expressing eYFP) and PV^+^ cells (expressing tdTomato) in acutely prepared *ex vivo* slices containing NAc (Figure 6B). We performed whole-cell current-clamp recordings of PV^+^ interneurons and neighboring MSNs (in NAc regions where dCA1 afferents overlapped with PV^+^ interneurons; Figures 6B and 6C) to investigate their response to dCA1 axon activation. Consistent with our findings above, both MSNs and PV^+^FSIs received functional inputs from dCA1 ChR2-eYFP axons, as revealed by the excitatory postsynaptic potentials (EPSPs) evoked in these cells upon NAc photo-stimulation (Figure 6D–6F). However, the light-evoked EPSPs in PV^+^FSIs were significantly larger in amplitude (Figure 6E) and had faster rise times (Figure 6F) than in MSNs. Moreover, PV^+^FSIs responded more robustly to successive (paired-pulse) stimulations of dCA1 inputs (Figure 6G). PV^+^FSIs, but not MSNs, discharged action potentials upon NAc photo-stimulation of dCA1 axons at increased light intensities (Figure 6H). This cell-type-specific spike firing was likely facilitated by the more depolarized resting membrane potential (Figure 6I) and the higher input resistance (Figure 6J) of NAc PV^+^FSIs, as compared to MSNs. None of the recorded neighboring NAc PV-negative interneurons responded to dCA1 inputs.

Together with the observation that *in vivo* stimulation of dCA1 inputs entrained NAc FSIs faster than MSNs (Figure 4H–4K), our findings raise the possibility that the combination of a strong dCA1 excitatory drive and a high intrinsic excitability of FSIs allows dCA1 PYRs to exert fine-grain control over MSN spiking using feedforward inhibition. To test this, we identified MSNs responding to stimulation of dCA1 ChR2-eYFP afferents as marked by optogenetically driven EPSPs while recorded in whole-cell current-clamp and switched to voltage-clamp recordings to determine the type of dCA1-driven postsynaptic currents. We found that dCA1 axon stimulation elicits both excitatory (EPSCs) and inhibitory (IPSCs) postsynaptic currents in all of these MSNs (Figures 6K and 6L). The co-existence of these two types of postsynaptic currents shows that the MSNs directly entrained by dCA1 PYR inputs also receive dCA1-driven feedforward inhibition.

These results establish that a subset of NAc PV^+^FSIs and MSNs receive functional dCA1 synaptic inputs. They further highlight that a combination of synaptic and cell intrinsic properties allows dCA1 PYRs to readily entrain NAc PV^+^FSIs and exert feedforward inhibitory control of their postsynaptic MSN targets.

### Intersectional and *Trans*-synaptic Strategy for Photo-silencing of dCA1-Connected NAc PV^+^FSIs

**Figure 7:**
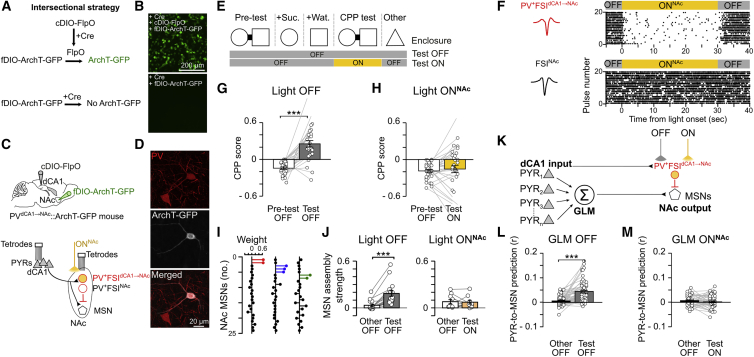
dCA1-Connected NAc PV^+^FSIs Are Required for the Retrieval of CPP Memory and Associated MSN Assemblies (A and B) Intersectional strategy to restrict the expression of ArchT-GFP to Cre-expressing cells (A). The translation of fDIO-ArchT-GFP construct was conditional to the sequential activity of Cre and FlpO recombinases, and validated in cultured HEK293T cells co-transfected with Cre and the cDIO-FlpO construct (B, top) or not (B, bottom). (C and D) This strategy was used *in vivo* for the *trans*-synaptic anterograde targeting of dCA1-connected NAc PV^+^ cells (C). PV^+^FSI^dCA1→NAc^ indicates a NAc PV^+^ interneuron that receives direct dCA1 inputs and that expresses ArchT (see red and white cell in confocal image of D), whereas PV^+^FSI^NAc^ indicates a neighboring interneuron that does not (see red-only cell in confocal image of D). PV^dCA1→NAc^::ArchT-GFP mice were implanted with tetrodes for dual-site monitoring of dCA1 and NAc ensembles, and optic fibers for light ON^NAc^ delivery. This strategy was also used for the *trans*-synaptic targeting of dSub-connected NAc PV^+^ cells (see Figures S7D–S7G). (E) Mice performed each day the 1-day CPP protocol and explored another enclosure (other), as before (Figures 2 and 3). Yellow light (20 × 30-s pulses over 15 min) was delivered during CPP test ON. Reward seeking was also measured during CPP tests (Figure S7B) and hedonic motivation further tested in another enclosure containing drops of sucrose in light ON^NAc^ (n = 11 mice tested; 10/10 collected drops of sucrose for all mice). Novel place preference was also tested in these mice (Figure S7C). (F) Examples of two NAc FSIs recorded in a behaving PV^dCA1→NAc^::ArchT-GFP mouse. Shown are average spike waveforms (left) and raster plots of spike discharge in relation to light ON^NAc^ (right). Note that the firing of the PV^+^FSI^dCA1→NAc^ cell (top) was suppressed during light delivery whereas the bottom FSI^NAc^ cell was not. (G and H) CPP score (mean ± SEM; n = 64 days from 11 mice) during pre-test and test (G: n = 30 light OFF; H: 34 ON^NAc^ days). (I) Examples of weight vectors showing NAc assembly patterns formed by short (25 ms) timescale co-activation of MSNs in the CPP apparatus. Each assembly is depicted in the form of a lollipop plot with MSNs ordered and color-coded to highlight those with coincidental spiking. (J) Activation strength of NAc assembly patterns detected from MSN co-activation in the CPP apparatus prior to test. Each pair of connected data points shows the average strength of an assembly pattern tracked during the Other enclosure and the CPP test (mean ± SEM; n = 16 light OFF, 10 ON^NAc^ patterns; 2.36 ± 0.43 patterns per mouse recording day; 13.91 ± 3.05 MSNs per mouse recording day). (K–M) Prediction of MSN spike trains from dCA1 PYR spike trains using generalized linear models (GLMs; K). In light OFF, GLMs trained on spike trains recorded prior to the test predicted MSNs during CPP test significantly above their prediction accuracy when applied in the Other enclosure (L; n = 58 MSNs from 30.25 ± 3.98 PYRs per GLM). Photo-silencing of PV^+^FSIs^dCA1→NAc^ dropped PYR-to-MSN prediction to the levels seen in the CPP-unrelated enclosure (M; n = 48 MSNs from 26.85 ± 4.12 PYRs per GLM). ^∗∗∗^p < 0.001, paired t tests.

We aimed to investigate whether dCA1 PYRs support CPP memory by influencing MSN spiking via dCA1-connected PV^+^FSIs. To address this, we implemented an optogenetic strategy to express ArchT selectively in NAc PV^+^ cells receiving dCA1 monosynaptic inputs (Figure 7A–7D). We based our approach on a double-conditional Boolean logic scheme of transgene expression, where ArchT-GFP is expressed in presence of both Cre and flippase (FlpO) recombinases within the same cell (Figures 7A and 7B). In our system, the two recombinases act in series: the presence of Cre allows the expression of FlpO, which in turn allows the expression of ArchT (Figure 7A). We packaged plasmids containing the Cre-dependent FlpO transgene (cDIO-FlpO) in a high-titer serotype 1 AAV vector capable of anterograde *trans*-synaptic transfer (Zingg et al., 2017), which we injected into the dCA1 of PV-Cre mice (Figure 7C). We then injected the other AAV harboring the FlpO-dependent ArchT-GFP (fDIO-ArchT-GFP) into the NAc of the same mice (Figure 7C). In these PV^dCA1→NAc^::ArchT-GFP mice, ArchT-GFP expression was successfully restricted to a subset of NAc PV^+^ interneurons, namely those connected to dCA1 (PV^+^FSI^dCA1→NAc^ cell in Figure 7D). This cell-type selective and input-defined targeting allows for testing whether PV^+^FSI^dCA1→NAc^ cells are required for CPP memory.

### dCA1 PYRs Orchestrate MSN Ensemble Firing Using NAc PV^+^FSI Postsynaptic Targets

**Figure S7:**
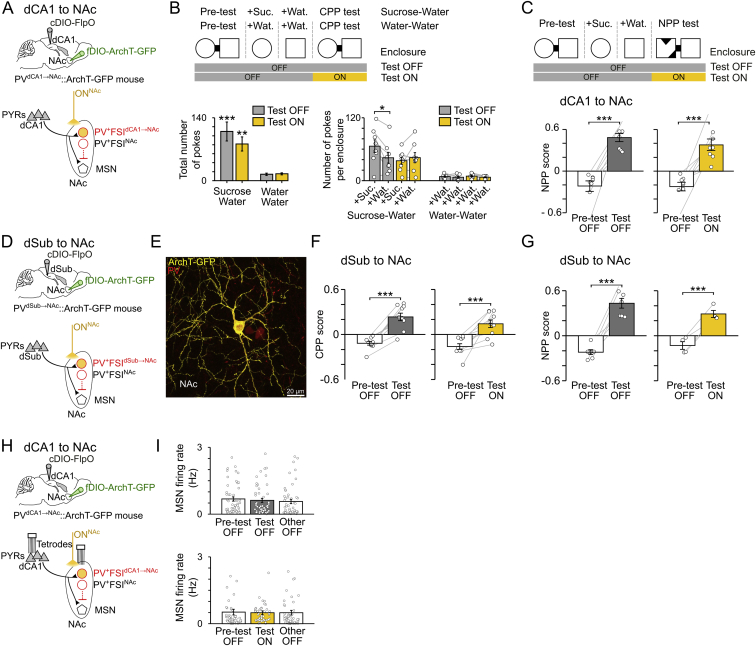
Complementary Analyses Related to the Optogenetic Silencing of dCA1- or dSub-Connected NAc PV^+^FSIs, Related to Figure 7 (A) Intersectional strategy used *in vivo* for the *trans*-synaptic anterograde transduction of dCA1-connected NAc PV^+^ cells with ArchT-GFP. The PV^dCA1→NAc^::ArchT-GFP mice were implanted with optic fibers above the NAc for light ON^NAc^ delivery. (B) Quantification of reward seeking behavior during the CPP test of mice exposed to sucrose and water during conditioning sessions, or only water (related to Figures 7G and 7H). Note that the number of pokes was unchanged between light OFF and ON conditions in mice conditioned to water only. (C) Mice were also tested for their ability to detect spatial novelty using the one-day novel place preference (NPP) task (as in Figure S4) during which the sucrose-paired enclosure of the conditioning phase was replaced by a novel enclosure during the NPP test. In light ON days, yellow light (20x30sec-pulses over 15min) was delivered during test. NPP score (mean ± SEM) measured during pre-test and test sessions (n = 9 light OFF NPP days and 8 light ON^NAc^ NPP days for PV^dCA1→NAc^::ArchT-GFP mice shown in Figures 7G and 7H). ^∗∗∗^p < 0.001, paired t tests. (D and E) Intersectional strategy used *in vivo* for the *trans*-synaptic anterograde transduction of dSub-connected NAc PV^+^ cells with ArchT-GFP. The PV^+^FSI^dSub→NAc^ refers to a NAc PV^+^ interneuron that receives direct dSub inputs and that expresses ArchT (see yellow and red cell in confocal image E), whereas PV^+^FSI^NAc^ refers to a neighboring interneuron that does not (see red-only cell in confocal image E). The PV^dSub→NAc^::ArchT-GFP mice (n = 8) were implanted with optic fibers above the NAc for light ON^NAc^ delivery. (F) CPP score (mean ± SEM) of PV^dSub→NAc^::ArchT-GFP mice measured during pre-test and CPP test (n = 10 light OFF CPP days and 10 light ON^NAc^ CPP days). ^∗∗∗^p < 0.001, paired t tests. (G) NPP score (mean ± SEM) of PV^dSub→NAc^::ArchT-GFP mice measured during pre-test and NPP test (n = 7 light OFF NPP days and 4 light ON^NAc^ NPP days). ^∗∗∗^p < 0.001, paired t tests. (H and I) PV^dCA1→NAc^::ArchT-GFP mice were implanted with tetrodes and optic fibers. The average firing rate of the recorded MSN population remained stable across the pre-test, CPP test and during the exploration in the Other (CPP-unrelated) enclosure during optogenetic silencing of dCA1-connected NAc PV^+^ cells (20x30sec-pulses over 15min in CPP test light ON^NAc^). See also Figures 7F and 7I–7M.

We implanted PV^dCA1→NAc^::ArchT-GFP mice with tetrodes for dual dCA1-NAc ensemble recordings and bilateral optic fibers above the NAc (light ON^NAc^; Figure 7C) to silence dCA1-connected PV^+^FSIs during CPP memory retrieval (Figures 7E and 7F). The strong place preference expressed by mice toward the sucrose-paired enclosure during light OFF test (Figure 7G) was no longer observed in days with optogenetic silencing of PV^+^FSIs^dCA1→NAc^ (Figures 7F and 7H). This memory impairment was reminiscent of that obtained during NAc photo-silencing of dCA1 PYR axons (Figure 2K) and was not caused by motivational or foraging deficits. Indeed, the same PV^dCA1→NAc^::ArchT-GFP mice still actively searched for rewards in the (no longer baited) containers during CPP test (Figures S7A and S7B); they also collected all drops of sucrose provided in another enclosure, while under identical light delivery condition. Moreover, mice remained able to detect spatial novelty during this optogenetic manipulation (Figure S7C).

We asked whether CPP selectively relies on the dCA1-connected set of PV^+^FSIs, or whether it could involve NAc FSIs receiving other inputs from the hippocampal formation. Building from our intersectional strategy, we observed that transducing dSub with the *trans*-synaptic approach in additional mice led to ArchT-GFP expression by a subset of NAc PV^+^FSIs (Figures S7D and S7E). However, the same experimental paradigm, this time applied to inactivate dSub-connected NAc PV^+^FSIs, did not prevent CPP behavior (Figure S7F). It also preserved unconditioned novel place preference (Figure S7G). These results show that the set of NAc PV^+^FSIs receiving inputs from dCA1, but not from dSub, plays a central role in CPP behavior; however, this dCA1-connected set of NAc PV^+^FSIs is dispensable for reward seeking, hedonic motivation, and spatial novelty detection (Figures S7A–S7C).

We finally investigated the circuit-level mechanism associated with spatial appetitive memory retrieval to understand the CPP impairment caused by the functional removal of dCA1-connected NAc PV^+^FSIs (Figure 7H). Silencing these PV^+^FSIs^dCA1→NAc^ did not significantly change the average firing rate of the MSN population (Figures S7H and S7I). We hypothesized that CPP relies on the ability of dCA1 PYRs to govern MSN ensemble spiking. We detected NAc assembly patterns formed by short (25-ms) timescale co-activation of MSNs in the CPP apparatus prior to the test (Figure 7I). We found that the successful CPP observed in light OFF (Figure 7G) was associated with the successful reinstatement of MSN assemblies (Figure 7J). In contrast, inactivating PV^+^FSIs^dCA1→NAc^ prevented both CPP (Figure 7H) and MSN assembly reinstatement (Figure 7J). To assess whether these effects related to the inability of dCA1 PYRs to orchestrate MSN firing during memory retrieval, we fitted generalized linear models (GLMs) to predict MSN spike trains from those of dCA1 PYRs during exploration of the CPP apparatus prior to the test (Figure 7K). These GLMs predicted MSN spike discharge more robustly when subsequently applied during light OFF CPP test, than when they were applied to spike trains monitored in another (CPP-unrelated) enclosure (Figure 7L). This showed that the firing relationships between dCA1 PYRs and NAc MSNs observed during the CPP procedure are selectively reinstated during memory retrieval. This was no longer the case during CPP test with NAc photo-silencing of PV^+^FSIs^dCA1→NAc^ (Figure 7M). In fact, the performance of these GLMs applied during the light ON^NAc^ test dropped to the low levels obtained when they were applied to predict MSN spiking in the other (CPP-unrelated) enclosure (Figure 7M). Together, these results reveal that dCA1 PYRs support CPP memory retrieval by orchestrating MSN assembly firing patterns via a select group of PV^+^FSIs.

## Discussion

In this study, we reveal a cross-circuit motif that directly connects PYRs of the dorsal hippocampal CA1 to both MSNs and a subset of PV^+^FSIs of the NAc to enable the behavioral readout of neuronal representations of space during appetitive memory retrieval.

### Neuronal Reinstatement and Behavioral Manifestation of a Memory

The cooperative spiking of hippocampal PYRs underpins the computation of information-representing assemblies thought to subsequently serve memory-guided behavior (Buzsáki, 2010, Moser et al., 2017). This important idea could lead to the assumption that the circuit-level expression of a memory representation and its behavioral manifestation are the same process. Here, we show that these are two dissociable, though complementary processes. Both dorsal hippocampus and NAc are critical to associate a specific environment with salient outcomes (Annett et al., 1989, Ferbinteanu and McDonald, 2001, Ito et al., 2008, Schacter et al., 1989). Accordingly, we found that suppressing dCA1 assemblies impaired place behavior during CPP memory retrieval. Importantly, preventing NAc reception of dCA1 information outflow similarly disrupted CPP behavior. However, in the second case, the impairment occurred despite the preserved reinstatement of the dCA1 assemblies important for the task. This functional dissociation between the neuronal reinstatement of a memory and its behavioral readout shows that the retrieval of hippocampal representations is necessary, but not sufficient, for the memory trace to serve an action that would increase the likelihood of finding reward outcomes. This has implications for the interpretation of the deficits observed in hippocampus-dependent tasks as these could arise from dysfunctional hippocampal processing of mnemonic information and/or altered information transmission to downstream targets for behavioral translation.

### Division of Behavioral and Mnemonic Labor across Hippocampal Projections

Previous studies have implicated neuronal spiking in the dorsal hippocampus-ventral striatum system in linking spatial context to natural reward outcomes (e.g., Lansink et al., 2012, van der Meer and Redish, 2009). The implementation of such a link remains uncertain. The dCA1 and the dSub are two important memory circuits (Cembrowski et al., 2018, Morris et al., 1990, Roy et al., 2017, van Strien et al., 2009) that both host PYRs sending excitatory inputs to NAc (French and Totterdell, 2002, Groenewegen et al., 1987). Our findings show that these pathways have distinct mnemonic contributions: the dCA1 projection to NAc is required for the retrieval of spatial appetitive memory, while dSub projection to NAc is dispensable. Furthermore, dCA1 inputs to dSub are dispensable for CPP memory retrieval but contribute to (unconditioned) spatial novelty detection, consistent with a role of dSub in the encoding of new spatial memories (Cembrowski et al., 2018, Morris et al., 1990). Our findings join recent work revealing dissociable information streams in the hippocampal formation (Cembrowski et al., 2018, Roy et al., 2017), which develop upon the canonical view of the hippocampal formation acting as a simple chain of connected circuits processing information in series. Instead, these results support the exciting perspective that the hippocampal formation hosts a complex set of cell-type- and circuit-selective pathways processing multiple, parallel information streams to serve distinct behavioral and mnemonic roles.

### dCA1 PYRs Drive Two NAc Neuronal Cell Types

The NAc receives various inputs, including those from the ventral hippocampus, amygdala, and prefrontal cortex, and projects to basal ganglia output nuclei, which in turn modulate cortical and brainstem motor centers (e.g., Ambroggi et al., 2008, Britt et al., 2012, French and Totterdell, 2002, MacAskill et al., 2012, O’Donnell and Grace, 1995, Scudder et al., 2018). Here, we report that dCA1 PYRs form synapses with the distal dendrites of MSNs, and that stimulating dCA1 inputs drives MSN responses. Our results complement studies showing that MSN activity correlates with activity of dorsal hippocampus PYRs and the observation that cocaine CPP relates to strengthened PYR-MSN coupling (Lansink et al., 2012, Sjulson et al., 2018). We also show that blocking the dCA1→NAc pathway increased the firing of some MSNs, while decreasing the firing of others. In addition to this bidirectional MSN firing modulation, inactivating the dCA1→NAc pathway decreased the firing of a subset of NAc FSIs. Interestingly, both the distribution of dCA1 PYR inputs and that of PV^+^FSI somata were biased to the rostral region of NAc. We did not observe such a rostro-caudal bias for other types of NAc interneurons. Yet, dCA1 inputs and the different interneuron types were more represented in NAc shell than core. Our findings are reminiscent of the rostro-caudal “keyboard-system” (Reynolds and Berridge, 2001), and suggest that, in addition to the distinction established between the shell and the core, the rostral NAc plays a central role in spatial appetitive behavior. We further found that stimulating dCA1 terminals elicited a fast response of some NAc FSIs, which can be explained by the triple (i.e., distal and proximal dendrites, and soma) dCA1 synaptic targeting as well as by the markedly different synaptic properties and higher excitability of those interneurons. By showing that dCA1 not only drives MSNs but also a subset of FSIs, our findings suggest that dCA1 orchestrates fine-grain MSN assembly spiking using a feedforward inhibition mechanism instantiated by a set of local PV^+^FSIs.

PV^+^ interneurons represent a small neuronal population in the striatum but are well positioned to majorly affect the MSN output (Gittis et al., 2010, Tepper et al., 2010). Consistent with this, we found that stimulating NAc PV^+^FSIs alters MSN population firing and that dCA1-connected PV^+^FSIs are required for dCA1-PYR-predicted MSN assemblies associated with CPP memory. The role of dCA1-connected PV^+^FSIs in CPP memory was not supported by any subset of FSIs, as dSub-connected NAc PV^+^FSIs remain dispensable for successful place-reward behavior. Thus, our data reveal that PYRs-to-MSNs, PYRs-to-PV^+^FSIs, and PV^+^FSIs-to-MSNs of the dCA1→NAc pathway are fundamental building blocks of a tripartite cross-circuit motif using excitatory drive and feedforward inhibition to serve the spatial guidance of adaptive behavior.

### Engaging NAc with Mnemonic Representations to Inform Behavior

The NAc has long been involved in reward-motivated behavior and described as an integrative site of inputs conveying information from various sources. Notably, a projection from the amygdala can provide the NAc with representations of discrete, reward-predicting cues (e.g., Ambroggi et al., 2008, Stuber et al., 2011). Here, we uncovered the dCA1→NAc pathway as pivotal for the behavioral translation of the neuronal representation of a spatial context during appetitive memory retrieval. The tripartite motif we define as the neural substrate for dCA1-NAc coupling could apply to other types of representations received by the NAc, where distinct sets of FSIs would support motifs engaging other inputs. These findings raise the exciting prospect that the combination of excitatory drives and feedforward inhibition in the NAc may be a general mechanism through which neuronal representations of distinct memory components may compete or cooperate to shape NAc assemblies and gain control over behavior.

## STAR★Methods

### Key Resources Table

**Table:** 

REAGENT or RESOURCE	SOURCE	IDENTIFIER
**Antibodies**		

All listed in Table S2		N/A

**Bacterial and Virus Strains**		

pAAV-EF1a-DIO-hChr2(h134R)-EYFP-WPRE-HGHpA	gift from K. Deisseroth	Addgene plasmid # 20298
pAAV-EF1a-fDIO-hChr2(H134R)-EYFP	gift from K. Deisseroth	Addgene plasmid # 55639
pPGKFLPobpA	gift from P. Soriano	Addgene plasmid # 13793
pAA-EF1a-cDIO-hChr2(E123T/T159C)-mCherry	gift from K. Deisseroth	Addgene plasmid # 35510
VPK-401 vector kit	Cell Biolabs, USA	N/A
AAV2-CAG-flex-ArchT-GFP	UNC Vector Core	N/A
AAV2-CAG-flex-GFP	UNC Vector Core	N/A
AAV2-EF1a-DIO-hChR2(H134R)-EYFP-WPRE	UNC Vector Core	N/A
AAV2-CamKII-hChR2(H134R)-EYFP	UNC Vector Core	N/A
AAV2-CAG-tdTomato	UNC Vector Core	N/A
VPK-401 vector kit	Cell Biolabs, USA	N/A
AAV2-EF1a-fDIO-ArchT-EGFP	UNC Vector Core (packaging)	N/A

**Experimental Models: Cell Lines**		

293T	ECACC	12022001

**Experimental Models: Organisms/Strains**		

CaMKII-Cre mice	https://www.jax.org	Stock #005359; RRID: IMSR_JAX:005359
Vglut2-Cre mice	https://www.jax.org	Stock #028863; RRID: IMSR_JAX:028863
PV-Cre mice	https://www.jax.org	Stock #008069; RRID: IMSR_JAX:008069
Ai9 mice	https://www.jax.org	Stock #007909; RRID: IMSR_JAX:007909
C57BL/6J mice	Charles River, UK	Strain code 632

**Software and Algorithms**		

Intan RHD2000	Intan Technologies, Los Angeles	http://intantech.com/RHD2000_evaluation_system.html
KlustaKwik	K. Harris	https://github.com/klusta-team/klustakwik/
Python 2.7	https://www.python.org	Python 2.7.13
Scikit-learn 0.18.1 (Python package)	https://scikit-learn.org/stable/	v0.18.1
Stats model (Python package)	http://www.statsmodels.org/stable/index.html	v0.8.0
Fiji	NIH	http://fiji.sc/
Stereoinvestigator	MBF Bioscience	Stereoinvestigator 9.0

**Other**		

12um tungsten wires	California Fine Wire	M294520
Optic fibers 10mm	Doric lenses, Québec, Canada	MFC_200/230-0.37_10mm_RM3_FLT
Optic fibers 25mm	Doric lenses, Québec, Canada	MFC_200/230-0.37_25mm_RM3_FLT
Optic fibers 29mm	Doric lenses, Québec, Canada	MFC_200/230-0.37_29mm_RM3_FLT
Optic rotary joint	Doric lenses, Québec, Canada	FRJ_1x2i_FC-2M3
64-channels amplifier	Sensorium, Charlotte, VT	EPA-6
Head-stage amplifier	Intan Technologies, Los Angeles	RHD2164
473nm diode-pumped solid-state laser	Laser 2000, Ringstead, UK	CL473-100-0
561nm diode-pumped solid-state laser	Laser 2000, Ringstead, UK	CL561-100-0
electron microscope	Philips	CM100
ultramicrotome	Leica Microsystems, Wetzlar, Germany	EM UC6
microtome	Leica Microsystems, Wetzlar, Germany	VT1000S

### Contact for Reagent and Resource Sharing

Further information and requests for resources and reagents should be directed to and will be fulfilled by the Lead Contact, David Dupret (david.dupret@pharm.ox.ac.uk).

### Method Details

#### Animals

We used male adult transgenic mice (3–8 months old) that were heterozygous for a transgene carrying the Cre recombinase, with expression driven by either the CaMKIIa promoter (RRID: IMSR_JAX:005359), the Vglut2 promoter (RRID: IMSR_JAX:028863) or by the PV promoter (RRID: IMSR_JAX:008069), as well as C57BL/6J mice (Charles River, UK). For *in vitro* experiments, NAc PV^+^ cells were also visualized using male adult transgenic mice obtained from the breeding of PV-Cre mice with Ai9 (tdTomato) reporter mice (RRID: IMSR_JAX:007909). All transgenic mice were maintained on a C57BL/6J background. Animals were housed with their littermates up until the first surgical procedure with free access to food and water in a dedicated housing room with a light/dark cycle (lights on 7 a.m. to 7 p.m.). All experiments involving animals were conducted according to the UK Animals (Scientific Procedures) Act 1986 under personal and project licenses issued by the Home Office following ethical review.

#### Cloning and plasmids

The pAAV-EF1a-fDIO-ArchT-EGFP plasmid vector (Figure 7) was constructed through the series of sub-cloning stages. First, the pAAV-Tre3G-ArchT-EGFP (Trouche et al., 2016) was used as a template to amplify two PCR fragments with the primers GGGCCCAGTAGCAGTGAGCAAGGGC and GGCGCGCCTTACTTGTACAGCTCGTCCATGC for the fragment A, primers GCTAGCGCCACCATGGACCCCATCGCTCT and GGGCCCCGGGGCTCGGGGGCCTCG for the fragment B. Both fragments were then sequentially inserted into pAAV-EF1a-double floxed-hChr2(h134R)-EYFP-WPRE-HGHpA (a gift from Karl Deisseroth, Addgene plasmid # 20298) using first restriction sites AscI and ApaI, then NheI and ApaI forming pAAV-Tre3G-DIO-ArchT-EGFP vector with the AscI restriction site. In the next step, the ArchT-EGFP ORF was sequentially inserted into pAAV-EF1a-fDIO-hChr2(H134R)-EYFP (a gift from Karl Deisseroth, Addgene plasmid # 55639) vector using first AscI and NheI restriction sites for the first fragment of the ORF, followed by non-directional insertion of a second portion of the ORF using the single NheI site. The recombinant AAV vector was packaged with AAV2 coat proteins (9.6e12 vg/ml, University of North Carolina). To make pAAV-Ef1a-cDIO-FLPo-Myc plasmid vector (Figure 7) FLPo recombinase ORF was amplified using pPGKFLPobpA vector (a gift from Philippe Soriano, Addgene plasmid # 13793) as a template with primers GCTAGCGCCACCATGGCTCCTAAG and GGCGCGCCTCACAAGTCCTCTTCAGAAATGAGCTTTTGCTCGATCCGCCTGTTGATGTAG. The PCR fragment was then inserted into pAA-EF1a-cDIO-hChr2(E123T/T159C)-mCherry (a gift from Karl Deisseroth, Addgene plasmid # 35510) using AscI and NheI restriction sites. The recombinant AAV vector was packaged in HEK293T cells with AAV1 coat proteins (1.6e13 vg/mL) using AAV-1 Helper Free Packaging System vectors, part of VPK-401 vector kit (Cell Biolabs Inc., USA). To assess their functionality both new vectors were tested for the recombinant protein expression in cultured HEK293T cells expressing Cre-recombinase.

#### Cell culture and transfection

HEK293T cells were grown in Dulbecco’s modified Eagle’s medium (Sigma), supplemented with 10% (v/v) FBS and 2 mM L-glutamine (all from Sigma) at 37°C in 5% CO_2_ at 100% humidity. For transient recombinant protein expression cells were transfected with PEImax (Polysciences Inc., USA) at 1.5e-3 mg/mL (DNA:PEI complex ratio 1:2) 48hrs prior to fixation and imaging (72hrs for virus production).

#### AAV vectors

AAV vector encoding Archaerhodopsin-T (ArchT) fused with the green fluorescent protein (GFP) under the control of a Cre-dependent CAG promoter (from E.S. Boyden; AAV2-CAG-flex-ArchT-GFP, 2.0E12 vg/mL, UNC Vector Core). AAV vector encoding the green fluorescent protein (GFP) under the control of a Cre-dependent CAG promoter (from E.S. Boyden; AAV2-CAG-flex-GFP, 2.0E12 vg/mL, UNC Vector Core). AAV vector encoding Channelrhodopsin-2 (ChR2) fused to an enhanced yellow fluorescent protein (EYFP) under the control of a Cre-dependent EF1a promoter (from Karl Deisseroth, AAV2-EF1a-DIO-hChR2(H134R)-EYFP-WPRE, 2.5E12 vg/mL, UNC Vector Core). AAV vector encoding ChR2-EYFP fusion under the control of the CaMKII promoter (AAV2-CamKII-hChR2(H134R)-EYFP, 1.5E12 vg/mL, UNC Vector Core). AAV vector encoding the fluorescent protein tdTomato under the control of a Cre-dependent CAG promoter (AAV2-CAG-tdTomato, 4.7E12 vg/mL, UNC Vector core). AAV vector encoding the FLPo recombinase under the control of a Cre dependent EF1a promoter (AAV1-EF1a-cDIO-FLPo-Myc; see section above for cloning), packaged in HEK293 cells with AAV1 coat proteins (1.6E13 vg/mL) using AAV-1 Helper Free Packaging System vectors VPK-401 vector kit (Cell Biolabs Inc., USA). AAV vector encoding the proton pump ArchT merged to an enhanced fluorescent marker EGFP under the control of flippase-dependent promoter (AAV2-EF1a-fDIO-ArchT-EGFP, see section above for cloning; packaged in UNC Vector Core, 9.6E12 vg/mL).

#### Surgery and tetrode with optic fiber implants

Mice were anaesthetized with isoflurane (0.5%–3.5% oxygen gas mixture 2 l/min) and buprenorphine (0.1 mg per kg of body weight) for stereotaxic injections. Viral injections were targeted unilaterally or bilaterally to dCA1 (−1.7mm and −2.3mm AP, +/− 1.7mm ML, −1.1mm DV; 450nl per site), dSub (−3.2mm and −3.4mm AP, +/− 1.5mm and ± 1.9mm ML, −1.55mm DV; 350nl per site) and/or NAc (+1.4mm AP, +/− 1.25mm ML, −4mm DV; +1.8mm AP, +/− 1.0mm ML, −3.8, −4.25 mm DV; 500nl per site) (Paxinos and Franklin, 2007). The viral vectors were delivered at a rate of 100nl/min using a glass micropipette lowered to the target site and that remained for 5min after injection before it was withdrawn. After 2 weeks of recovery mice were implanted with a microdrive containing ten or twelve independently movable tetrodes and/or one or two optic fibers (Doric lenses, Québec, Canada).

#### Tissue processing for electron microscopy

ArchT-GFP expression was assessed in CamkII-Cre mice injected in the right dCA1 with the CAG-flex-ArchT-GFP viral construct (Figures 1, 5, S1, and S6). One month post injection, CamKII^dCA1^::ArchT-GFP mice were anaesthetized and perfuse-fixed with 4% paraformaldehyde and 0.1% glutaraldehyde for electron microscopic analysis. Coronal sections (50μm) were cut using a vibrating blade microtome (VT1000S; Leica Microsystems, Wetzlar, Germany). All sections were washed 3 times in phosphate buffer (PB) saline (PBS) and placed into a cryoprotectant (0.05M PB, 25% sucrose, 10% glycerol) for a minimum of 2 hr before freeze-thawing. Sections were blocked (10% NGS Normal goat serum in PBS, NGS-PBS; Vector Laboratories, Peterborough, UK; Cat# S-1000, RRID:AB_2336615) and washed (2% NGS) prior their incubation in primary antibodies. Sections were incubated in a primary antibody against GFP as a marker for dCA1 terminals in the NAc (1:1000, Monoclonal rat IgG2a, Clone GF090R, 04404-84, Nacalai Tesque) and a primary antibody against parvalbumin (PV) as a marker for GABAergic fast-spiking interneurons in the NAc (1:1000, Polyclonal, 195004, Synaptic Systems; Guinea Pig). GFP was revealed using a peroxidase method with diaminobenzidine (DAB) as the chromogen, PV was revealed using the silver-intensified immuno-gold method (Doig et al., 2010). Sections were then incubated for 4hrs in a biotinylated (Vector BA-9400; goat anti-rat, dilution 1:300) and a gold-conjugated (1.4 nm colloidal gold, Nanoprobes#2055; goat anti-guinea pig, dilution 1:300) secondary antibody. To reveal PV with silver intensification of the conjugated gold particles, sections were washed in PBS and then in acetate buffer (0.1M sodium acetate 3-hydrate, pH 7.0–7.5). Silver reagent (1ml; HQ Silver kit, Nanoprobes) was added to each section and allowed to react for 4–5 min in the dark, washed three times each in acetate buffer and then PBS. The peroxidase method was then used to reveal GFP; sections were incubated in avidin-biotin-peroxidase complex (ABC) for at least 90 minutes, and then washed in Tris buffer (TB; 0.05M, PH 7.4) before incubation in diaminobenzidine (DAB; 0.025% in TB) for 15 minutes. Hydrogen peroxide (0.03%) was added to start the reaction, after 5-7 min the reaction was stopped by washing in TB. All sections were then washed three times in 0.1M PB (pH 7.4). Sections were post-fixed in osmium tetroxide (1% in PB; Oxkem, Oxford, UK) for 10min. They were then washed in 0.1M PB and dehydrated in an ascending series of ethanol dilutions (15 min in 50% ethanol, 35min in 70% ethanol which included 1% uranyl acetate (TAAB, Reading, UK), 15 min in 95% ethanol, and twice 15 min in absolute ethanol). Following absolute ethanol, sections were washed twice in propylene oxide (Sigma) for 15 min and placed into resin (Durcupan ACM, Fluka, Gillingham, UK) and left overnight at room temperature. The resin was then warmed to reduce viscosity and sections were placed on microscope slides, a coverslip applied and the resin cured at 65°C for about 70h.

#### Electron microscopic analysis

All sections were examined in the light microscope and areas from the NAc, where GFP-DAB axon was present, were cut from the slide, glued to the top of a resin block and trimmed with razor blades. For each animal 3 blocks (small regions of axon-dense tissue) containing NAc were examined; 2 blocks were examined from the NAc shell, and 1 from the NAc core. Regions were examined from at least 2 rostral-caudal sections. Serial sections (∼50 nm thick; silver/gray), were then cut using an ultramicrotome (Leica EM UC6, Leica Microsystems) and collected on pioloform-coated, single-slot copper grids (Agar Scientific, Stansted, UK). The sections were then lead-stained to improve contrast for electron microscopic examination. A Philips CM100 electron microscope was used to examine the ultrathin sections. GFP-positive structures were systematically analyzed in one of the serial sections on an electron microscopic grid. At a magnification where it is not possible to visualize synapses an area was chosen at random, the magnification was then increased and the first structure positively labeled for GFP which was forming a synapse was digitally recorded (Gatan multiscan CCD camera, Gatan, Oxfordshire, UK). Analyses were performed at a minimum of 5 μm from the tissue-resin border (i.e., the surface of the section). The maximum distance from the tissue-resin border examined was determined by the penetration of the gold conjugated antibody together with the angle at which the tissue-resin was sectioned, and was therefore variable. GFP-positive structures forming synapses were identified and imaged, in this way, continuing systematically in straight lines across the section, keeping the identified GFP-positive structures central within the image frame. This systematic process was continued until at least 100 GFP-positive structures forming synapses were identified and imaged per animal. GFP-synapse forming terminals were sampled from 3 blocks (regions) per animal (2 shell and 1 core), and a minimum of 2 grids per block were examined (a total of 353 synapses, n = 3). For each image the postsynaptic target of the GFP-positive terminal was identified as being i) a spine of an MSN, ii) aspiny unlabeled dendrite, or iii) immunogold-labeled (PV+) dendrite. For immunogold-labeled structures, the criterion for an immunopositive structure was five or more silver-intensified immunogold particles. MSN somata were identified by a thin cytoplasm relatively lacking in mitochondria and endoplasmic reticulum, a uniformly spherical cell body and nucleus that further lacks in indentations (Bolam et al., 1983). Digital images were analyzed using the publicly available software, ImageJ. Images were adjusted for contrast and brightness using Adobe Illustrator and Photoshop (Version CS5, Adobe Systems Incorporated, San Jose, CA).

#### Immunohistochemistry

All mice were anaesthetized with pentobarbital following completion of the experiments and transcardially perfused with PBS followed by 4% PFA / 0.1% glutaraldehyde in PBS solution. Brains were extracted and chronic implants were removed. The brains were kept in 4% PFA for at least 24 h before slicing and coronal sections (50 μm thick) were stored in PBS-azide. Optionally, sections were prepared for the immunohistochemistry procedures: free-floating sections were rinsed extensively in PBS with 0.25% Triton X-100 (PBS-T) and were blocked for 1-2 h at room temperature in PBS-T with 10% normal donkey serum (NDS). Sections were then incubated overnight at room temperature with primary antibodies diluted in 3% NDS blocking solution (See Table S2). After further washing using PBS, sections were incubated overnight at room temperature in PBS-T containing a mixture of secondary antibodies (See Table S2) diluted in the blocking solution. After further washing in PBS, sections were mounted on slides, coverslipped with Vectashield mounting medium (Vector Laboratories, Cat. No. H-1000) and stored at 4°C. See Table S2.

#### Stereological sampling of interneuron subtypes in NAc

Four coronal sections containing the NAc corresponding to Bregma positions +1.7, +1.5, +1.0 and +0.7 (Paxinos G. and Franklin K.B.J., 2007) were selected from each mouse for analysis. These encompassed the greater part of the rostro-caudal extent of the nucleus. The coronal sections were initially viewed at a 5X magnification in a Zeiss Imager M2 epifluorescence microscope. Interneurons of each subtype were imaged in the NAc at a 20X magnification, using the optical disector method. This method involves taking 10 z-stacked images 1 μm thick in each 50 μm thick section, from a 2 μm position below the surface of the section. Cells were only counted if they came into focus through the 10 μm optical slice. The position of each interneuron type was then plotted using Stereoinvestigator 9.0. The density of each interneuron subtype within the NAc of each coronal section was determined by dividing the number of interneurons by the respective volume in which they were counted. The volumes were determined by measuring the cross-sectional areas of the sections and multiplying them by the sample depth (10 μm). The densities from the matched Bregma sections for three mice were averaged. The mean densities were then compared across the rostro-caudal extent of the NAc in the 4 positions to Bregma.

#### One-day place preference tasks (CPP and NPP)

The CPP apparatus consisted of two square-walled (46 cm × 46 cm × 38 cm) enclosures with distinct inside building block configurations. The two enclosures were connected via a bridge (8-cm length, 7-cm width) during pre-test, CPP test and mismatch test. All mice were handled for at least 3 days before the first CPP experiment. Mice were food restricted (to ∼90% body weight) and were selected after performing a 1-day CPP procedure consisting of 4 sequential phases: pre-test, place conditioning to sucrose (+S), to water (+W) and test. Mice were subjected to a novel CPP configuration (i.e., a novel building block configuration) on each testing day. During pre-test, mice explored the entire apparatus for 15min, after which their baseline (spontaneous) preference for one of the two enclosures was determined. Next, the bridge was removed for the conditioning session, and mice explored their non-preferred enclosure containing drops of 20% sucrose diluted in water (2x10min sessions, 10x10 μL drops per session). Next, mice explored their preferred enclosure containing drops of water (2x10min sessions, 10x10 μL drops per session). One hour later, the behavioral expression of the CPP memory was assessed by allowing the mice to explore the entire apparatus for 15min (CPP test). Optionally, light was delivered *in vivo* throughout the implanted optic fibers during test (see Light delivery section). We calculated a place preference score for each mouse during both pre-test and test sessions as the difference between the time spent in the enclosure paired with sucrose minus that paired with water during conditioning over their sum. Mice also explored another, CPP-unrelated, open-field enclosure (“Other”) on each CPP day. Reward-seeking behavior was assessed by the number of pokes to the empty dispensers in each enclosure during CPP test (light OFF and ON) (Figures S3F–S3H and S7B). To test the memory for reward (sucrose) experience, the poke analysis was also performed in mice exposed to water drops only during conditioning sessions (Figure S7B). To examine the ability to detect spatial novelty, mice were also subjected to a novel place preference (NPP) test for which the sucrose-paired enclosure of the CPP apparatus was replaced by a novel enclosure (Figures S4, S7C, and S7G). To test their hedonic motivation, PV^dCA1→NAc^::ArchT-GFP mice were also exposed for 15min in light ON condition to another open-field enclosure containing 10x10 μL drops of sucrose and the number of drops collected was counted afterward.

#### In vivo light delivery

In order to optogenetically silence neuronal cell bodies or their axonal projections, a diode-pumped solid-state laser (Laser 2000, Ringstead) was used to feed implanted optic fibers with yellow (561nm) light (∼15–20mW input power; ∼9–11mW output power) either to dCA1 (Figure 2; 20x30s pulses over 15min with probabilistic inter-pulse intervals of 10-20 s), dSub (Figures 3 and S4; a 1min pulse in the home-cage followed by a 10min pulse from the beginning of the test session) or NAc (Figures 2, 3, 4, 7, S3, S4, S5, and S7; a 1min pulse in the home-cage followed by a 10min pulse from the beginning of the test session or 20x30s pulses over 15min with probabilistic inter-pulse intervals of 10-20 s as described in the text). In order to optogenetically stimulate dCA1 ChR2-expressing axons in the NAc, a 473nm diode-pumped solid-state laser (Laser 2000, Ringstead) was used to deliver trains of 5ms-long blue light (∼15-20mW) pulses to the NAc of CamKII^dCA1^::ChR2-eYFP mice (Figure 4H–4K). To optogenetically silence dCA1- or dSub-connected NAc PV interneurons, 561nm light was delivered in the NAc (20x30s pulses over 15min with probabilistic inter-pulse intervals of 10-20 s) of PV^dCA1→NAc^::ArchT-GFP and PV^dSub→NAc^::ArchT-GFP mice (Figures 7 and S7). In order to optogenetically identify NAc PV-expressing interneurons, a 473nm diode-pumped solid-state laser (Laser 2000, Ringstead) was used to deliver blue light to the NAc (10ms pulses over 20min with probabilistic inter-pulse intervals of 1–4 s) of PV^NAc^::ChR2-eYFP mice (Figure 5).

#### Brain slice electrophysiology

Acute brain slices containing the NAc were made from adult mice 6–8 weeks post-virus injection. Mice were aneasthetised with isoflurane and then decapitated. Brain was removed and coronal 350-400μm slices were cut using a vibrating microtome (Microm HM650V). Slices were prepared in artificial cerebrospinal fluid (aCSF) containing (in mM): 65 Sucrose, 85 NaCl, 2.5 KCl, 1.25 NaH_2_PO_4_, 7 MgCl_2_, 0.5 CaCl_2_, 25 NaHCO_3_ and 10 glucose pH 7.2-7.4, bubbled with carbogen gas (95% O_2,_ 5% CO_2_). Slices were immediately transferred to a storage chamber containing aCSF (in mM): 130 NaCl, 3.5 KCl, 1.2 NaH_2_PO_4_, 2 MgCl_2_, 2 CaCl_2_, 24 NaHCO_3_ and 10 glucose pH 7.2–7.4, at 32°C and bubbled with carbogen gas until used for recording. Slices were transferred to a recording chamber and continuously superfused with aCSF bubbled with carbogen gas with the same composition as the storage solution (32°C and perfusion speed of 2 ml/min). Whole-cell current-clamp and voltage-clamp recordings were performed using glass pipettes, pulled from standard wall borosilicate glass capillaries and containing for current-clamp experiments (in mM): 110 potassium gluconate, 40 HEPES, 2 ATP-Mg, 0.3 Na-GTP, 4 NaCl and 4 mg/ml biocytin (pH 7.2–7.3; osmolarity, 290–300 mosmol/l) and for voltage-clamp recordings (in mM): 120 cesium gluconate, 40 HEPES, 4 NaCl, 2 ATP-Mg, 0.3 Na-GTP, 0.2 QX-314 and 4 mg/ml biocytin (pH 7.2–7.3; osmolarity, 290 –300 mosmol/L). Recordings were made using Multiclamp 700B amplifiers and acquired using WinWCP software (University of Strathclyde, UK).

#### Slice stimulation and recording protocols

Recording of resting membrane potential was performed in whole-cell current-clamp mode (Table S1). Hyperpolarising and depolarising current steps were used to assess the intrinsic properties of the recorded neurons including input resistance and action potential frequency. Action potential frequency was used to assist with the classification of neurons as fast-spiking interneurons or medium-sized spiny neurons. Dorsal hippocampal input to NAc neurons was studied by optically stimulating ChR2-eYFP expressing dCA1 axons in NAc. Fibers were activated every 5–10 s for up to 20 times and excitatory postsynaptic potentials (EPSPs) were recorded in patched NAc neuron. For voltage-clamp experiments, MSNs that showed a dCA1-evoked EPSP were clamped at −75 mV to record excitatory postsynaptic currents (EPSCs), and subsequently clamped at 0 mV to record inhibitory postsynaptic currents (IPSCs). The same light stimulation protocol was used for voltage-clamp as with current clamp recordings. Photo-activation of ChR2 dCA1 axons was achieved using widefield 2–3ms duration light pulses via a TTL triggered CoolLED pE-300 system on the microscope (CoolLED, Andover, UK). The steady-state light power at the tissue was measured using a PMD100D power meter and photodiode sensor (Thorlabs). Stimulation strength was at 0.8mW (and high stimulation strength was at 1.6 mW) at the tissue. For paired-pulse stimulation, two pulses of 2-3ms were consecutively given at 50ms interval and repeated every 10 s for up to 20 times.

#### Analysis of slice recordings

Data were analyzed offline using Igor Pro (Wavemetrics). Synaptic inputs from dCA1 were assessed by averaging the 10-20 sweeps of optically evoked single spike or trains of spike stimulation and detecting EPSPs, EPSCs or IPSCs. These were defined as upward deflections of more than 2 standard deviations above baseline. The input resistance was calculated by dividing the membrane potential observed after hyperpolarizing the membrane potential with 300pA current. The analysis of EPSP/EPSC/IPSC kinetics (peak amplitude, duration and rise time) was performed on average synaptic responses. The peak amplitude was measured as the amplitude of the EPSP/EPSC/IPSC relative to baseline. The duration was taken as the time between the start and the end of the EPSP. The rise time was taken as the time between 20% of the maximum amplitude and 80% of the maximum amplitude of the EPSP. The paired-pulse ratio was taken as the amplitude of the second EPSP divided by the amplitude of the first EPSP.

#### Histological analyses on slices

Following whole-cell patch-clamp recording, slices were fixed in 4% paraformaldehyde in 0.1M phosphate buffer (PB; pH 7.4). Biocytin-filled cells were visualized using streptavidin fluorescent-conjugated antibodies (ThermoFisher Scientific, Cat#S32351) performed using standard procedures. All sections were pre-incubated in 10%–20% normal goat serum (NGS; Vector Laboratories; Cat# S-1000, RRID:AB_2336615) in PBS containing 0.3% Triton-X (PBS-Tx) for more than 1h at RT. Slices were co-stained in 1:100,000 DAPI in PBS-Tx to facilitate the delineation of brain structures and co-incubated with the following antibodies. Endogenous fluorescence was amplified with primary and secondary antibodies listed in Table S2. All sections were mounted in Vectashield (Vector Laboratories, Cat. No. H-1000) and images were captured with a LSM 710 (Zeiss) confocal microscope using ZEN software (Zeiss).

#### In vivo ensemble recordings

After the recovery period of at least one week following the surgical injection/implantation procedures, mice were daily familiarized to the experimental paradigm, including handling, connection to the recording system and exploration of open-fields. Thereafter, on the morning of each recording day, the local field potential (LFP) signals obtained from each tetrode was used to guide its optimal positioning within the dCA1 pyramidal layer (Csicsvari et al., 1999) and/or within the NAc (Berke, 2009) in search of multi-unit spiking activity. Tetrodes were then left in position for ∼1.5-2h before recordings started. Ensemble recordings were performed during active exploratory behavior in the CPP apparatus and/or an open field environment concomitant or not with *in vivo* light delivery for optogenetic manipulations. At the end of each recording day, tetrodes were raised to avoid possible mechanical damage overnight.

#### Multichannel data acquisition

The signals from the electrodes were amplified, multiplexed, and digitized using a single integrated circuit located on the head of the animal (RHD2164, Intan Technologies; gain x1000). The amplified and filtered (0.09Hz to 7.60kHz) electrophysiological signals were digitized at 20kHz and saved to disk along with the synchronization signals from the position tracking and the laser activation. To track the location of the animal three LED clusters were attached to the electrode casing and captured at 25 frames per second by an overhead color camera. The signal was transmitted offline and aligned with the registered analog position tracking and laser pulse time stamps.

#### Spike detection and unit isolation

The electrophysiological traces were subsequently band pass filtered (800Hz to 5kHz) and single extracellular discharges detected through thresholding the RMS power spectrum using a 0.2ms sliding window. Detected spikes of the individual electrodes were combined per tetrode. To isolate spikes putatively belonging to the same neuron, spike waveforms were first up-sampled to 40kHz and aligned to their maximal through. Principal component analysis was applied to these waveforms ± 0.5ms from the through to extract the first three or four principal components per channel, such that each individual spike was represented by 12 waveform parameters. An automatic clustering program (KlustaKwik, http://klusta-team.github.io) was run on this principal component space and the resulting clusters were manually recombined and further isolated based on cloud shape in the principal component space, cross-channels spike waveforms, auto-correlation histograms and cross-correlation histograms. All sessions recorded on the same day were concatenated and clustered together. Each cluster used for further analysis showed throughout the entire recording day stable cross-channels spike waveforms, a clear refractory period in its auto-correlation histogram, well-defined cluster boundaries and an absence of refractory period in its cross-correlation histograms with the other clusters.

#### Neuronal assembly analysis

Firing patterns of co-active dCA1 pyramidal cells or co-active NAc MSNs were detected using an unsupervised statistical framework based on independent component analysis (Figures 2, 7, and S2) (Lopes-dos-Santos et al., 2013). Spikes discharged by each neuron were counted in 25ms time bins and standardized (z-scored, i.e., the activity of each neuron was set to have null mean and unitary variance), to avoid a bias toward neurons with higher firing rates. The neuronal population activity was represented by a matrix in which each element represents the z-scored spike count of a given neuron within a given time bin. Then, we extracted assembly patterns in a two-step procedure. First, the number of significant co-activation patterns embedded within the neuronal population was estimated as the number of principal components of the activity matrix with variances above a threshold derived from an analytical probability function for uncorrelated data. Finally, we applied independent component analysis to extract the assembly patterns from the projection of the data into the subspace spanned by the significant principal components (i.e., each assembly was captured by an independent component). The activity of each assembly at time *t* was computed as:

$$A_{t}=Z_{t}^{T}PZ_{t}$$

where $Z_{t}$ is a vector carrying the instantaneous z-scored rate of each neuron at time *t*, P is the projection matrix (outer product) of the corresponding independent component, and *T* is the transpose operator. Note that $A_{t}$ is the squared projection of $Z_{t}$ onto the component that represents the assembly. This projection represents the similarity between the independent component and the population rate at a given time bin. In order to eliminate the contribution of single neurons to the assembly activity, we set the main diagonal of P to zero. Moreover, to achieve a high temporal resolution, the spike train of each neuron, sampled at 20kHz was convolved with a Gaussian kernel (and then z-scored). The assembly pattern activations used to compute assembly maps were defined using peaks in the expression strength (Figure S2). To assess the enclosure-specificity of assembly patterns, we compared each pattern detected during the CPP pre-test with the set of patterns detected during the test session in the same CPP enclosure and with the set of patterns detected during exposure to another (“Other”) enclosure. Pattern detection in CPP tests was performed using the entire session (that is, using both light OFF and ON epochs). The similarity of two assembly patterns was quantified by a similarity index equal to the absolute value of the inner product of their weight vectors.

#### Physiological classification of NAc neurons

The band-pass filtered (800Hz to 5kHz) and up-sampled (40kHz) signal traces of the spikes discharged by each individual NAc cell were aligned and averaged to obtain the mean waveform for each channel of the recording tetrode. We used the mean waveform with the highest amplitude among the channels of a given tetrode for subsequent classification of that cell. The extracted mean spike waveform, designated as the function of time w(t) (with w(t = 0) the global minimum of the function) was parameterized by three numerical measures: the duration of its first positive moment (or “peak”), the duration of its second peak and the spike symmetry (Figure S5A). The duration of the first peak was defined as the time interval between the global minimum of w(t) (i.e., the spike trough; t = 0) and the second zero intercept of the waveform (w(t) = 0) to its left. We defined similarly the duration of the second peak to the right of the spike trough. The spike symmetry was defined as the amplitude of the first peak (the local maxima to the left of the spike trough) over the sum of the amplitudes of the first and the second peaks. The three clusters defining MSN, FSI and TAN cells were identified by scaling the distribution of each feature and applying the k-means algorithm (Figure S5A). The clusters were then confirmed with a density-based spatial clustering of applications with noise (DBSCAN) and a hierarchical clustering algorithms.

#### dCA1-NAc spike timing relationships

We computed the discharge probability of NAc FSIs (Figure 4F; Original spikes) and MSNs (Figure 4G; Original spikes) around the spikes discharged by PYRs during active behavioral epochs marked by dCA1 theta oscillations. To disentangle the contribution of theta coherence between dCA1 and NAc and potential short-latency dCA1-NAc interactions, we also computed control cross-correlations by shifting each dCA1 PYR spike to a random theta cycle while preserving its original theta phase (Figures 4F and 4G; Shifted spikes). Therefore, control spike trains have the same theta phase histogram as their original version. Figures 4F and 4G display mean and confidence interval of shift controls drawn from 1000 surrogates.

#### dCA1-to-NAc Spiking Ensemble Prediction

We computed generalized linear models (GLMs) to test whether the spike train of each recorded MSN could be predicted from the activity of the simultaneously recorded dCA1 PYRs. To do so, we binned the ongoing firing activity of both PYRs and MSNs to obtain binned spike trains that span the exploration of the CPP apparatus and the “Other” enclosure. The time bins we used as single observations for this prediction were defined by the theta cycles recorded from the pyramidal layer of the hippocampal dCA1 since these provide natural time windows for the expression of dCA1 assemblies (Buzsáki, 2010). More specifically, the firing activity of each PYR or MSN cell was represented by a vector in which each element is the number of spikes discharged by that cell within a given theta cycle. We fitted a GLM to predict the activity of MSN *j* by the PYR population during the exploration of the CPP apparatus before the test as follow:

$$MSN_{j}\left(\theta \right)=\beta_{0}+\sum_{i}\beta_{PC_{i}}PC_{i}\left(\theta \right)+error$$

Then, the obtained model was applied to predict MSN activity during the CPP test or the exploration of the Other enclosure. In order words, MSN activity during the CPP test or the exploration of the Other enclosure was predicted by the model obtained from previous exploration of the CPP apparatus. We quantified the accuracy of each model as the linear correlation between the observed firing activity of each MSN and its values predicted by the model output. Regressions were implemented with the scikit-learn package (https://scikit-learn.org/stable/) in Python 2.7 (https://www.python.org/downloads/release/python-2714/).

### Quantification and Statistical Analysis

The means of two sets of observations were compared with a two-sample t test or, if they were obtained from the same set of assembly patterns, with a paired t test, unless stated otherwise. Reported group data are mean ± SEM, unless stated otherwise.
