# Supplementary material for: A Hippocampus-Accumbens Tripartite Neuronal Motif Guides Appetitive Memory in Space
Source: Cell. 2019 Mar 7;176(6):1393–1406.e16. doi: 10.1016/j.cell.2018.12.037 (PMC6424821; doi:10.1016/j.cell.2018.12.037)
Supplement: Document S1. Tables S1 and S2 [file mmc1.pdf]

Cell, Volume *176*

Supplemental Information

A Hippocampus-Accumbens Tripartite Neuronal Motif Guides Appetitive Memory in Space

Stéphanie Trouche, Vadim Koren, Natalie M. Doig, Tommas J. Ellender, Mohamady El-Gaby, Vítor Lopes-dos-Santos, Hayley M. Reeve, Pavel V. Perestenko, Farid N. Garas, Peter J. Magill, Andrew Sharott, and David Dupret

Table S1. Intrinsic membrane properties of NAc neurons (*related to Figure 6*).

	dCA1-responding	Not dCA1-responding	p
**MSN**			
Resting membrane potential (mV)	-78.8 ± 1.8	-78.4 ± 2.3	0.98
Input resistance (MΩ)	98.1 ± 11.0	88.5 ± 9.1	0.69
Spike rate (+300pA) (Hz)	18.7 ± 2.9	20.0 ± 2.6	0.82
First ISI (ms)	29.9 ± 4.1	31.2 ± 7.1	0.65
Second ISI (ms)	28.2 ± 3.1	36.3 ± 8.0	0.61
First spike amplitude (mV)	73.1 ± 3.6	71.4 ± 3.6	0.78
Second spike amplitude (mV)	56.7 ± 4.0	58.9 ± 5.3	0.82
First spike duration (ms)	1.9 ± 0.1	2.1 ± 0.2	0.36
Second spike duration (ms)	3.0 ± 0.3	2.9 ± 0.2	0.91
**PV^+ interneuron**			
Resting membrane potential (mV)	-71.0 ± 2.7	-64.0 ± 3.1	0.20
Input resistance (MΩ)	159.2 ± 28.4	161.0 ± 55.1	0.79
Spike rate (+60pA) (Hz)	57.5 ± 18.6	41.1 ± 16.1	0.28
First ISI (ms)	14.9 ± 3.9	17.8 ± 2.2	0.63
Second ISI (ms)	12.1 ± 3.3	10.5 ± 2.7	0.79
First spike amplitude (mV)	80.5 ± 4.5	99.5 ± 14.9	0.28
Second spike amplitude (mV)	73.0 ± 4.7	87.2 ± 15.7	0.57
First spike duration (ms)	0.8 ± 0.1	1.3 ± 0.3	0.85
Second spike duration (ms)	0.9 ± 0.2	1.4 ± 0.3	0.41
**non-PV^+ interneuron**			
Resting membrane potential (mV)	n/a	-65.0 ± 1.4	
Input resistance (MΩ)	n/a	289.1 ± 28.1	
Spike rate (+300pA) (Hz)	n/a	12.1 ± 2.6	
First ISI (ms)	n/a	29.9 ± 14.4	
Second ISI (ms)	n/a	29.4 ± 8.2	
First spike amplitude (mV)	n/a	68.5 ± 5.8	
Second spike amplitude (mV)	n/a	48.0 ± 4.0	
First spike duration (ms)	n/a	1.7 ± 0.4	
Second spike duration (ms)	n/a	2.0 ± 0.4	

Data are mean ± SEM; statistical comparisons by Mann-Whitney

Table S2. List of antibodies (*related to STAR Methods*)

	Primary antibodies			Secondary antibodies		
**Figure**	**Antibody**	**Dilut.**	**Source**	**Antibody**	**Dilut.**	**Source**
3	Rb-α-wfs1	1:500	Proteintech Group Cat# 11558-1-AP, RRID:AB_2216046	Dk α-Rb Cy3	1:1000	Jackson ImmunoResearch Labs Cat# 711-165-152, RRID:AB_2307443
5	Rb α-CR	1:1000	Synaptic Systems 214102 RRID:AB_2228331	Dk α-Rb 488	1:250	Thermo Fisher Scientific Cat# A-21206, RRID:AB_2535792
5, 6	Gp α-PV	1:1000	Synaptic Systems 195004 RRID:AB_2156476	Dk α-GP AMCA<br>Dk α-GP Cy3<br><br>Gt α-Gp 647	1:1000<br>1:1000<br><br>1:500	Jackson ImmunoResearch Labs Cat# 706-155-148, RRID:AB_2340458<br>Thermo Fisher Scientific Cat# A-21450, RRID:AB_2735091
S6	Rb α-NOS	1:1000	Millipore Cat# AB5380, RRID:AB_91824	Dk α-Rb 488	1:500	Thermo Fisher Scientific Cat# A-21206, RRID:AB_2535792
S6	Ms α-ChAT	1:500	Gift C. Cozzari	Dk α-Ms 647	1:250	Jackson ImmunoResearch Labs Cat# 715-605-151, RRID:AB_2340863
1, 4, 5, S1, S6	Rat α-GFP	1:1000	Nacalai Tesque Cat# 04404-84, RRID:AB_10013361	Gt α-Rat (Biotinylated)	1:300	Vector Laboratories Cat# BA-9400, RRID:AB_2336202
5, S6	Gp α-PV	1:1000	Synaptic Systems 195004 RRID:AB_2156476	Gt α-Gp (1.4nm gold-conjugated)	1:300	Nanoprobes
6	Chk α-GFP	1:1000	Aves Labs Cat# GFP-1020, RRID:AB_10000240	Gt α-Chk 488	1:500	Thermo Fisher Scientific Cat# A-11039, RRID:AB_2534096
6	Rat α-RFP	1:1000	ChromoTek Cat# 5f8-100, RRID:AB_2336064	Gt α-Rat 555	1:500	Thermo Fisher Scientific Cat# A-21434, RRID:AB_2535855

Abbreviations: Dilut.: Dilution; AMCA: 7-amino-4-methylcoumarin-3-acetic acid; ChAT: choline acetyltransferase; Chk: chicken; CR: calretinin; Dk: donkey; GFP: green fluorescent protein; Gt: goat; Gp, guinea pig; NOS: neuronal isoform of nitric oxide synthase; PV: parvalbumin; Rb, rabbit; RFF: red fluorescent protein.
